# Is air pollution exposure linked to household income? Spatial analysis of Community Multiscale Air Quality Model results for Madrid

**DOI:** 10.1016/j.heliyon.2024.e27117

**Published:** 2024-02-24

**Authors:** Richard J. Hewitt, Eduardo Caramés, Rafael Borge

**Affiliations:** a Institute of Economy, Geography and Demography, Centre for Human and Social Sciences, National Research Council (CSIC), C/ de Albasanz, 26, 28037, Madrid, Spain; b Transport and Territory Research Group (t-GIS), Department of Geography, Madrid Complutense University, 28040, Madrid, Spain; c Observatorio para una Cultura del Territorio, C/ del Duque de Fernán Núñez, 2,1, 28012, Madrid, Spain; d Escuela Técnica Superior de Ingenieros Industriales, Departamento de Ingeniería Química Industrial y del Medio Ambiente, Universidad Politécnica de Madrid (UPM), C/ José Gutiérrez Abascal, 2, 28006, Madrid, Spain

**Keywords:** Environmental justice, Household income, Air pollution, CMAQ model, GWR, Bivariate correlation analysis

## Abstract

This study explores the potential correlation between income and exposure to air pollution for the city of Madrid, Spain and its neighboring municipalities. Madrid is a well-known European air pollution hotspot with a high mortality burden attributable to nitrogen dioxide (NO_2_) and fine particulate matter (PM_2.5_). Statistical analyses were carried out using electoral district level data on gross household income (GHI), and NO_2_ and PM_2.5_ concentrations in air obtained from a mesoscale air quality model for the study area. We applied linear regression, bivariate spatial correlation analysis, spatial autoregression and geographically weighted regression to explore the relationship between contaminants and income. Three different strategies were adopted to harmonize data for analysis. While some strategies suggested a link between income and air pollution, others did not, highlighting the need for multiple different approaches where uncertainty is high. Our findings offer important lessons for future spatial geographical studies of air pollution in cities worldwide. In particular we highlight the limitations of census-scale socio-economic data and the lack of non-model derived high-resolution air quality measurement data for many cities and offers lessons for policy makers on improving the integration of these types of essential public information.

## 1 Introduction

### 1.1 Air pollution state of the art and policy challenges

Outdoor air pollution is known to cause serious health impacts and to increase mortality and is considered by the World Health Organization (WHO) as the world's greatest threat to environmental health [1,2]. The proportion of the global population living in urban areas is on the rise and expected to increase further in future [3]. Population exposure, and consequently increased mortality in urban areas, is therefore likely to rise unless action is taken. Improving air quality in cities is therefore an important priority for international bodies like the WHO and the European Union. Increasingly the issue is being taken up by city authorities, (e.g. Refs. [[4], [5], [6]]), though less so in the Global South [7]. Specifically, air pollution refers to high ambient air concentrations of specific contaminants, usually from motorized vehicles and the burning of fossil fuels for energy and heat [8,9]. Particulate matter (PM), nitrogen dioxide (NO_2_) and ground-level ozone (O_3_), are regarded as especially dangerous for human health [9]. Given the great diversity of heating and transport systems in cities, as well as climatic factors, ambient air concentrations vary widely in time and space. In European cities, high population densities in suburban areas combined with high *per capita* vehicle ownership rates leads to traffic congestion at key entrances, exits and intersection points into cities (traffic-related hotspots: see, e.g. Ref. [10]). This gives rise to significant concentrations of NO_2_ and PM_2.5_ at particular locations [11]. The typical daily meteorological cycle also plays an important role in ambient pollution concentrations, for example, the phenomenon of the late evening NO_2_ peak [12,13].

At the same time, most cities today tend to be spatially segregated by socioeconomic group. Property prices tend to be higher in well-connected areas with high quality amenities [14], and lower income residents tend to be forced out of these areas to the urban periphery [15]. Poor air quality and its related drivers (proximity to roads and industry) is likely to be a factor in residents’ neighborhood choice, with the least well-off being least able to choose [16]. In this sense, many studies have sought to investigate whether there is a relationship between socioeconomic factors and exposure to air pollution, reflected by various indicators, e.g. immigration status, race, age profile, or educational level [[17], [18], [19]]. In general terms, the Environmental Kuznets Curve (EKC) states that environmental degradation increases as a result of economic growth, but then declines after a high level of *per capita* income is reached. However [20], has noted that while concentrations of some pollutants have declined in some high-income countries, others have increased, which implies that the relation between income and pollution is neither direct nor statistically generalizable. At the urban neighborhood scale, the question is much more difficult to resolve. Ref [21] reported a relationship between deprivation (% unemployment, % low education, % manual & temporary workers) and air pollution concentrations for the city of Barcelona. However, these authors interpolated air pollution concentrations based on a few widely dispersed monitoring stations, which is likely to have affected the reliability of concentration values in between stations. Ref [22] investigated the relationship between PM_2.5_ concentrations collected by air quality monitors and the fractions of the non-white population and population living below the poverty line in Pittburgh, USA. No correlation was found between higher presence of these socioeconomic factors and higher PM_2.5_ concentrations. However, PM_2.5_ data were obtained from a small number of stations, and spatial autoregressive effects were not included in the analysis.

Most existing studies are heavily reliant on data from air pollution monitoring stations or sensors, which tend to have sparse coverage and large areas of missing data. To address this problem, sophisticated modelling approaches have been developed and deployed worldwide. Air quality models, such as Community Multiscale Air Quality Models (CMAQ), offer an opportunity to examine this variability at medium-high spatial resolution (e.g. Ref. [23]). At present however, few studies have employed these models for detection of relationships between air pollution and socioeconomic factors like deprivation, unemployment or income at the local level. In this study we address this research gap using simulated concentrations output from a CMAQ model for two airborne pollutants, NO_2_ and PM_2.5_, and household income data at the census tract level to explore the relationship between lower incomes and higher concentrations of air pollution in the city of Madrid and its neighboring municipalities. Our study is one of the first to employ modelled air pollution data at medium-high spatial resolution for the analysis of socio-economic variables, and the approach we present is likely to be of interest to future research on this timely and important topic.

### 1.2 The Madrid Study area

**Fig. 1:**
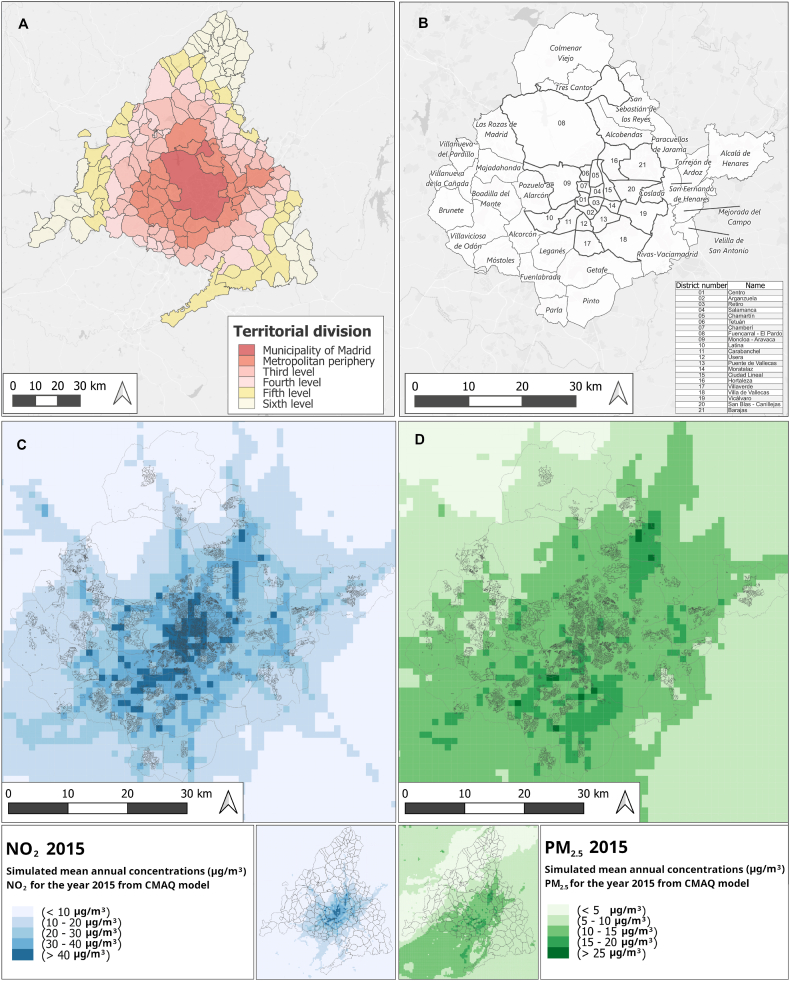
A: The municipality of Madrid (red) and B, suburban municipalities comprising the metropolitan area. Source: Own work based on data from Spanish National Geographical Institute; classifications according to Ref. [24]; C: Map of simulated concentrations of NO_2_ and D: Map of simulated concentrations of PM_2.5_, classified according to World Health Organisation (WHO) limit values (Source: CMAQ Model, see e.g. Ref. [1]). (For interpretation of the references to colour in this figure legend, the reader is referred to the Web version of this article.)

The city of Madrid is an urban municipality in centre of Spain with an area of 604.3 km^2^ and over 3 million inhabitants (Fig. 1A and B).

An estimated 2.5 million vehicles start or finish in the city of Madrid on a typical weekday, with around 40 million km being driven on a typical day in the city [1]; this inevitably generates major concentrations of airborne contaminants. Madrid is a well-known European air pollution hotspot, with, according to one study, the highest mortality burden attributable to nitrogen dioxide (NO_2_) in Europe [25]. Air pollution in Madrid causes an estimated 88 deaths per year from particulate matter (PM) and 519 from NO_2_, which is equivalent to 4 deaths per 100,000 inhabitants in the first case, and 23 deaths per 100,000 cases in the second [1]. Within the municipality of Madrid it has been estimated that 74.4% of all local NO_2_ emissions (i.e. those arising from within the city itself), are attributable to road traffic [26]. Air pollution concentrations in Madrid are highly spatially and temporally variable, due to traffic concentrations at key entrances and exits as well as local meteorological conditions. Like many modern cities, the core urban area comprising the municipality of Madrid is surrounded by adjacent commuter towns which are home to large populations attracted by easy access to the city centre and comparatively lower property prices. These adjacent municipalities comprise the metropolitan periphery (Fig. 1, [24]), and are important centers of population and industry, but also strongly residential in character, and highly unequal socioeconomically. Two such municipalities, Pozuelo de Alarcón and Boadilla del Monte, to the west of the city, contain several census districts with gross household incomes among the highest in Spain (all 129,750€) ([27], data from 2019). Four others, Alcalá de Henares to the east of the city, and Parla, Getafe and Leganés to the south each contain a census district with household incomes among the lowest in Spain (all <25,000€) ([27], data from 2019). For these reasons, we chose a study area including the whole metropolitan area, incorporating both the municipality of Madrid and the metropolitan periphery described above (Fig. 1A and B). The study area therefore includes income disparities that are among the largest in Spain, as well as key urban pollution hotspots.

## 2 Methods

Data comprised two different groups: 1) modelled air pollution concentrations, and 2) gross household income data in Euros collected between 2015 and 2018.

### 2.1 Air pollution data

For air pollution, we used spatially resolved simulated concentrations of nitrogen dioxide (NO_2_) and particulate matter less than 2.5 μm in aerodynamic diameter (PM_2.5_), output from a Eulerian photochemical air quality model known as the Community Multiscale Air Quality Model (CMAQ) for Madrid. These two pollutants have already been used in other articles on social inequality [28] and are the most relevant regarding health impacts of air pollution specifically in Madrid [1]. The CMAQ model estimates the concentration in μg/m^3^ of NO_2_ and PM_2.5_ for the year 2015 for the whole of the Community of Madrid. These data take the form of a square grid of 1 km^2^ cells, in which each cell in the grid is a unique georeferenced polygon object in vector GIS format with attached attribute containing the estimated concentration value. The model has been subject to extensive operational validation and is known to offer a consistent performance throughout the study area, suggesting that the model is able to accurately represent pollution spatial gradients that are essential for the validity of our research. A detailed discussion of operational evaluation procedures and model performance is provided in Supplementary Material, Part 1.

Fig. 1 (C,D) shows the spatial distribution of both pollutants in the study area as estimated by the CMAQ model. For NO_2_ (Fig. 1C) concentrations tend to be found in the centre of the study area in the city of Madrid and decrease as the distance from the centre increases. The lowest NO_2_ concentrations are found in the northern part of the Madrid metropolitan area and in the east of the municipality of Alcalá de Henares. For PM_2.5_ (Fig. 1D), the central area shows concentrations of between 10 and 15 μg/m^3^, with some areas in the northeast and south showing higher concentrations, between 15 and 25 μg/m^3^. The high estimated concentration values in the south correspond to the Madrid district of Villa de Vallecas, and the adjacent metropolitan municipalities of Getafe, Pinto and Leganés. Although these concentrations are not found in the census districts, which may be because these emissions come from road traffic or from industries located in these areas. As with NO_2_ concentrations, the lowest values are found in the northwest and east of the municipality of Alcalá de Henares. In view of the limited coverage provided by air quality monitoring stations, these simulated concentrations represent the best available information on the spatial extent of these two pollutants. Nonetheless, as with any simulation model output, these data sources should be approached with caution, as a result of the unavoidable uncertainty and error arising from the modelling process.

### 2.2 Household income data

**Fig. 2:**
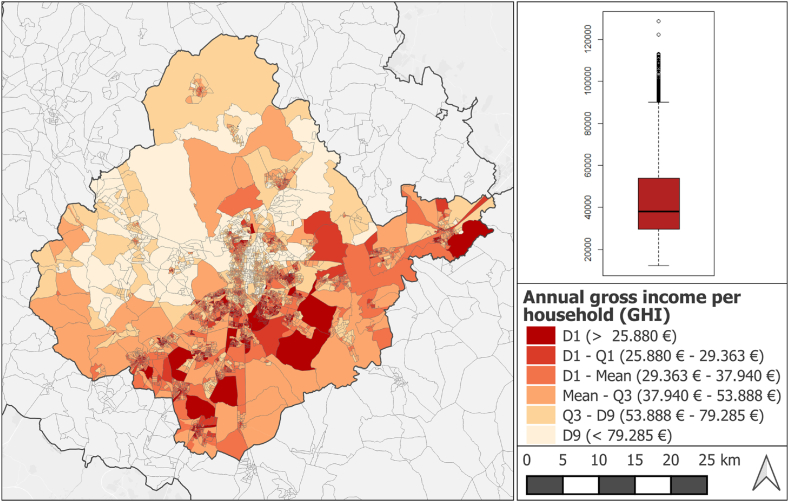
A (left) Distribution of mean annual gross income per household (GHI) in the metropolitan area and the city of Madrid [Source: Own work based on data from the National Geographical Institute (IGN) (urban residential areas) and National Statistics Institute (INE)]. B (right) Boxplot of GHI in the study area.

For household incomes, we used the Atlas of Household Income of the National Statistical Institute of Spain [27]. We extracted gross household income (GHI) data at the level of the census tract, the highest resolution data currently publicly available for the year 2015, to match the date for which simulated air pollution data were available (Fig. 2). Where data were not available for the year 2015, the nearest available date was chosen (2016, 2017 or 2018). Census tracts are the statistical unit inferior to the municipality that is the basis of the statistical operations of National population censuses. Every municipality is divided into one or more census tracts and there is no part of any municipality that does not belong to a census tract. Census tracts vary in size according to the number of inhabitants, with the most populous municipalities having many more census tracts than those with few inhabitants. Madrid municipality has over 2400 census tracts, the peripheral municipality of Boadilla del Monte (Fig. 1) has 27, and Zarzalejo, a rural municipality, has just one. In our study area, the smallest census tract is just 0.6 ha, the largest 1762 ha, though large census tracts are very uncommon (median approx 4.4 ha).

GHI in the study area varies between 12,153€ and 128,571€, with median 37,940€. With SD = 22,282€ and mean = 45,574€ this gives a coefficient of variation of 48.89%. Fig. 2 shows the high degree of variability between incomes in the study area, with a noticeable dividing line from lower left to upper right. The highest incomes (upper 10%, lightest colour) are uniformly located to the north of this line, while the lowest incomes (lower 10%, dark red) are all found to the south of this line. In the core city at the centre of the study area, census tracts are smaller, reflecting higher population density, and the pattern is more heterogenous, with a mixture of lower and higher income neighbourhoods.

### 2.3 Data preparation and statistical analysis

To enable analysis to be effectively carried out in a way that accounted for the mismatch in spatial units (census tracts for GHI, km^2^ for simulated contamination concentrations) we adopted three different strategies, as follows.

#### 2.3.1 Strategy 1: aggregation of GHI for each km^2^ in the air quality model grid

First, we transformed the vector polygon coverage of GHI by census tract into raster format (rasterization). We used a zonal statistics operation in GIS to extract and summarize the pixel level GHI data obtained from the rasterization operation for each km^2^ of the CMAQ model grid. The zonal statistics operation produced four outputs for each km^2^: 1) mean GHI within each km^2^; 2) maximum GHI within each km^2^; 3) minimum GHI within each km^2^; 4) total GHI within each km^2^. We then carried out ordinary least squares regression (OLS) for all kms^2^ using simulated NO_2_ concentration for the year 2015 as the dependent variable (y), and each of the four variants of GHI as the independent variable (x). The process was repeated using simulated mean annual PM_2.5_ concentration for the year 2015 as the dependent variable (y). Since only minimum GHI (minGHI) produced a significant response for p < 0.01 with >10% of the variance explained (for both NO_2_ and PM_2.5_) and the GHI variants are clearly not independent from each other, a multiple regression model was not used. To ensure normal distribution of the residuals – a key assumption of linear regression – a log transformation was performed on both dependent and independent variables.

To account for the Modifiable Aerial Unit Problem [29], where statistical information can be shown to depend on the size of the zone in which it is sampled or reported, as well as uncertainty derived from the rasterization operation [30], we: 1) rasterized the GHI vector layer at four different resolutions – 48 m, being the most appropriate cell size for the GHI vector layer according to Piwowar's rule (reported by Ref. [31]),^1^ 100 m, 200 m and 500 m; 2) we summarized the zonal statistics from GHI raster maps at 48 m, 100 m, 200 m and 500 m resolutions using a larger vector grid obtained by grouping pairs of individual km^2^ together to create a 4 km^2^ grid. In this way, both the effect of cell resolution of the rasterization of the GHI data as well as the effect of the size of the reporting units were tested. In total, we carried out 64 OLS operations: aggregated to 1 km^2^ and 4 km^2^ (2 variants) from 48 m,100 m, 200 m and 500 m pixel size raster (4 variants), for mean, max, min and total (4 variants), for NO_2_ and PM_2.5_ (2 variants) (2 x 4 x 4 x 2 = 64). Sample sizes used in the OLS regressions comprised 1880 data points in the case of data aggregated to the 1 km^2^ grid (i.e. the total number of 1 km^2^ grid squares in the study area), and 504 data points for the data aggregated to the 4 km^2^ grid (i.e. the total number of 4 km^2^ grid squares in the study area). Full analysis results are provided in Supplementary Material, Part 2.

As noted above, for both the simulated concentrations of NO_2_ and PM_2.5_ (Fig. 1) and GHI (Fig. 2) values appeared to cluster together in particular locations. This phenomenon, known as spatial autocorrelation, is virtually ubiquitous in geographical data, but can be problematic if not accounted for in regression models. Regression analysis of spatially autocorrelated data leads to low precision (high variance, giving poor model fit) and Type 1 errors (claiming a correlation where no such correlation exists, or claiming no correlation where a correlation does exist) [32]. Spatial autocorrelation was formally confirmed for NO_2_ and PM_2.5_ and min, max, mean and sum GHI using a Moran's I test [33]. To understand the implications of spatial autocorrelation across the study area the relationship between NO_2_ and min GHI and PM_2.5_ and min GHI was explored using Geographically Weighted Regression (*GWmodel* package in R). GWR is a well-known technique designed to overcome the limitations of global regression approaches where variables are highly spatially autocorrelated, and has been used in many comparable studies, especially in public health (e.g. Ref. [34]). In Strategy 1, the GWR approach described by Ref. [35] was followed. The approach involves dividing the study area up into local circular windows known as kernels, in which the diameter of the circle is known as the bandwidth and carrying out individual local regressions within each kernel. Data points with the kernel are weighted according to their distance away from the centre of the kernel, giving them declining influence in the regression equation as distance increases [35]. Finding the right kernel bandwidth size for the scale of the phenomena to be analysed is a key problem in GWR. In Strategy 1, we used a trial-and-error approach in which GWR analysis was carried out for 10,000, 5,000 and 2,500 m bandwidths, and standard error was computed for each set of results using the bootstrapping technique [35].

#### 2.3.2 Strategy 2: aggregation of simulated contaminant concentrations from the air quality model for each census tract containing GHI statistics

Strategy 2 is effectively the reverse of Strategy 1, in that we obtained aggregate contaminant data by census tract rather than aggregate income data by km^2^. Beginning with a vector polygon coverage of GHI by census tract, we carried out an intersection operation (QGIS) between the census tracts layer and the km^2^ grid data for contamination. We then calculated sum, maximum, minimum and mean contaminant values for all of the km^2^ grids intersecting each census tract. We then carried out OLS for all census tracts using each of the four variants of simulated NO_2_ concentration (sum, mean, min and max) for the year 2015 as the dependent variable (y), and GHI as the independent variable (x), with each OLS model having a sample size of 3839 (i.e. the total number of census tracts in the study area). These OLS models had no explanatory power (R^2^ < 0.01). We then carried out spatial regression in GeoDa software [36] using a Spatial Autoregessive Model (SAR), which confirmed the result of the OLS model. The SAR model showed NO_2_ values in the neighborhood of each census tract to be a very strong predictor of NO_2_ values in a census tract and GHI to be an extremely weak predictor of NO_2_ values in a census tract. Given this clear and unequivocal negative result, no further analyses were carried out under Strategy 2.

#### 2.3.3 Strategy 3: Downscaling GHI data to residential land use and subsequent aggregation of simulated contaminant concentrations from the air quality model for urban residential areas containing GHI statistics

The first task for Strategy 3 was to try to eliminate the discrepancies in census tract size by assigning GHI data to a more realistic spatial unit. Where urban residential areas are concentrated only in one corner of a large census tract as is frequently the case, the income values which must logically relate to the urban residential areas are applied to the whole census tract, masking any spatial variation in regression relationships between income and contamination. To correct for this, we assigned GHI values only to urban residential areas (rather than to the entire census tract) using a spatial intersection operation. To find the simulated values for mean annual concentrations of our two contaminants at the level of the residential area, we used a spatial join operation between the urban residential areas with attached GHI data (layer 1) and km^2^ grids for NO_2_ (layer 2) and PM_25_ (layer 3). The statistical summary operation in the GIS software was used to generate minimum, maximum, sum and mean values of mean annual concentrations of NO_2_ and PM_25_ for each urban residential feature.

First, as a preliminary exploratory step, we tested the power of mean annual gross income per household (GHI) to predict the simulated concentrations of each pollutant. To achieve this, we carried out ordinary least squares regression (OLS) using simulated minimum, maximum, mean and total annual NO_2_ concentration for the year 2015 in each urban residential area and GHI as the independent variable (x), obtaining 4 regression equations, with each OLS model having a sample size of 8600 data points. The process was repeated using simulated mean annual PM_2.5_ concentration for the year 2015 as the dependent variable (y). Due to the small size of the urban residential area polygons, minimum, maximum, and mean values were very similar in most cases, and regression models for these variants were therefore also similar. To account for spatial autocorrelation (see above for Strategy 1), we explored the spatial variance in the relationship between simulated mean annual concentrations of the two contaminants and GHI using spatial bivariate Local Moran's I (BiLISA) using the GeoDa software package, and GWR in R (spgwr package). BiLISA explores the degree of correlation between two spatially explicit variables by accounting for the variance between the value of each variable in each spatial unit and their values in the neighboring spatial unit (defined as contiguous using the Queen's case) – the so-called spatially lagged variable. The analysis identifies clusters along four axes for each variable x and y: low x with low y neighbours (LL), low x with high y neighbours (LH), high x with low y neighbours (HL) and high x with high y neighbours (HH). BiLISA is a feature of several recent studies, and has been used, for example, by Ref. [37] to identify local tendencies in the location of different types of accommodation in tourist cities; by Ref. [38] to explore the spatial relationship between ecosystem services and urbanisation, and by Ref. [39] to reveal the spatially varying relationship between local *per capita* GDP and air quality.

## 3 Results

### 3.1 Strategy 1: OLS results

Global level OLS regression indicated a negative correlation between level of household income and exposure to both NO_2,_ and PM_2.5_. Although the model fit did vary depending on the resolution and grid size chosen, the correlation was clearly present at all resolutions and both grid sizes. The strongest association (highest coefficient of determination R^2^ and lowest residual standard error RSE) between income and air pollution was found for minimum gross household income (MinGHI) and NO_2,_ and MinGHI and PM_2.5_. The global regression models explained between 10% and 20% of the variance for MinGHI and NO_2_, and between 12% and 19% of the variance for MinGHI and PM_2.5_, depending on resolution and grid size chosen. Standard residual error varied between 0.55 and 0.58 for MinGHI and NO_2_ and between 0.28 and 0.30 for MinGHI and PM_2.5_. Having established that the correlations were present at all resolutions and both grid sizes, the 1 km × 1 km grid was used with the 48 × 48 m resolution GHI data for the GWR analysis, being the original model grid size and the recommended resolution according to Piwowar's rule [31]. As the best performing variable in the OLS analysis, only MinGHI was retained as the independent variable in the GWR analysis.

### 3.2 Strategy 1: GWR results

**Fig. 3:**
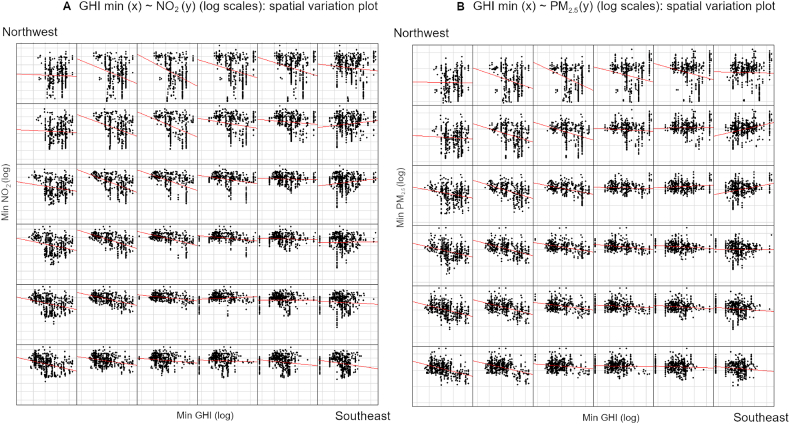
A (left): coplot for NO_2_ indicating variation in regression relationships across the study area; B (right): coplot for PM_2.5_ indicating variation in regression relationships across the study area.

To explore the degree of variation across the study area implied by the spatial autocorrelation test, we used the *coplot* function described by Ref. [35] to split the study area into equal sized panels and visualize the relationships in each part of the study area using the separate panels. The coplots (Fig. 3) show a steeper regression line in the central northern part of the study area (top centre), and a much shallower one elsewhere, with the tendency being flat or even slightly reversed at the easternmost extreme (right centre). This indicates a steeper rate of decrease in concentrations of contaminants as minimum income increases in the north of the study area, and a relationship which is either absent or undetectable in the east. There is little difference in the pattern between either of the two contaminants. Also notable in both plots is the smaller spread of the y-axis data, corresponding to NO_2_ (Fig. 3A), and PM_2.5_ (Fig. 3B), in the centre of the study area where nearly all contamination concentrations are high, compared especially to the upper panels where there is more variation in contamination concentrations, with a larger number of lower values indicating cleaner air to the north of the study area.

The coplots confirmed the impression of high spatial heterogeneity indicated by the Moran's I test. The GWR results allowed this phenomenon to be explored in more detail, indicating a much greater range of variation in the regression coefficients that could be seen from the global OLS results (Table 1).

**Table 1:** Results of GWR analysis under Strategy 1, in comparison with results of OLS regression. All values are base-10 log transformed.

Analysis	Variables (y ∼ x)	Coefficient	Mean Fit	Mean CI (lwr)	Mean CI (upr)	Coefficient Min	Coefficient median	Coefficient Max
Global OLS NO_2_	NO_2_ ∼ MinGHI	−0.489	2.882	1.768	3.996	_	_	_
GWR NO_2_ BW 10000	NO_2_ ∼ MinGHI	_				−0.602	−0.226	0.396
GWR NO_2_ BW 5000	NO_2_ ∼ MinGHI	_				−0.809	−0.072	0.895
GWR NO_2_ BW 2500	NO_2_ ∼ MinGHI	_				−1.160	0.006	1.512
Global OLS PM2.5	PM_2.5_ ∼ MinGHI	−0.254	2.396	1.820	2.972	_	_	_
GWR PM_2.5_ BW 10000	PM_2.5_ ∼ MinGHI	_				−0.291	−0.1	0.304
GWR PM_2.5_ BW 5000	PM_2.5_ ∼ MinGHI	_				−0.455	0.011	0.594
GWR PM_2.5_ BW 2500	PM_2.5_ ∼ MinGHI	_				−0.678	0.056	0.531

(CI = Confidence Interval).

While the global OLS regression equation estimated a coefficient value (m in the linear regression equation y = mx + c) across the whole study area of −0.489 (NO_2_) and −0.254 (PM_2.5_), GWR coefficient estimates unsurprisingly vary much more widely. For every one unit of change to the variable MinGHI, at bandwidth 10000 m, the mean increase in the response variable NO_2_ or PM_2.5_ varies from −0.602 to 0.396 (NO_2_) and −0.291 to 0.304 (PM_2.5_) (Table 1). As the table shows, the variation increases as bandwidth decreases. Though these values are not intuitively meaningful because of the log transformation, the change of sign indicates an important difference in the regression line depending on the specific locality investigated. In other words, in some parts of the study area, the correlation is negative (less income = more contamination), while in others the correlation is positive (more income = more contamination). This analysis provides more detail than we could obtain from the coplots, and reliable quantification of the degree of variation in the relationships explored.

**Fig. 4:**
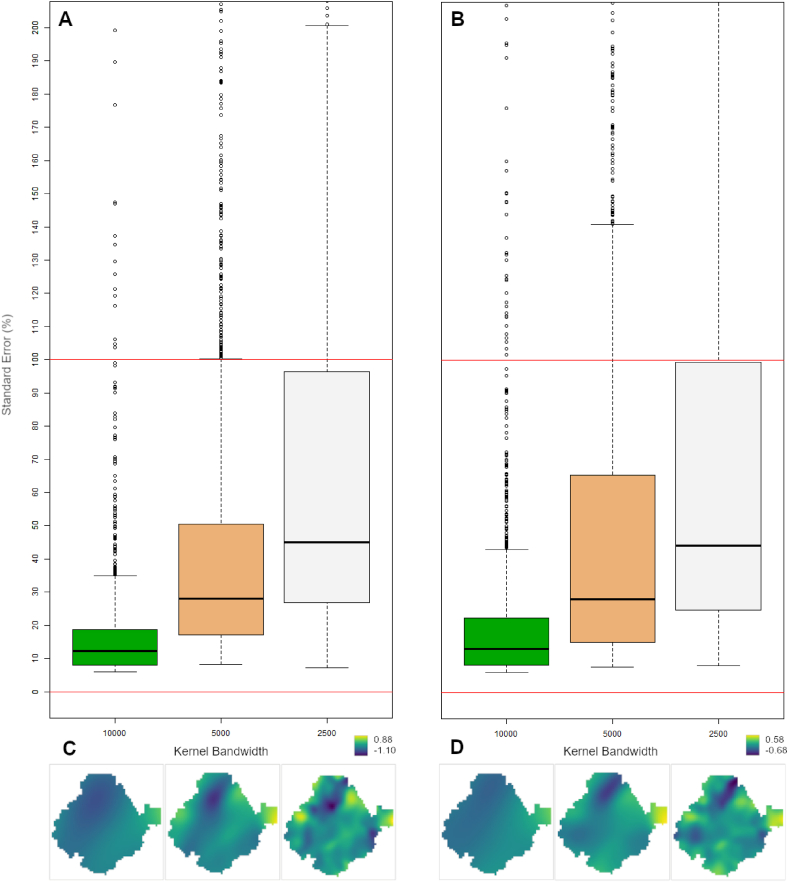
*Top*: Boxplots showing *S*tandard Error (SE) estimates for GWR results for NO_2_ (A) and PM_2.5_ (B), for each bandwidth as a percentage of the coefficient estimates. Horizontal red lines indicate SE of 0% and 100% of coefficient estimates. Note that for the PM_2.5_ GWR analysis results, the percentage error is greater than for NO_2_ for both 10000 m and 2500 m bandwidths*; Bottom:* GWR results for NO_2_log ∼ minlog (C) and PM_2.5_log ∼ minlog (D), for each bandwidth. (For interpretation of the references to colour in this figure legend, the reader is referred to the Web version of this article.)

The plots of these results (Fig. 4, bottom: C, D) clearly showed variation in the relationship between contaminant concentrations and minimum income, with increasing minimum income leading to a steeper fall off in contamination in the northern and central parts of the study area. Although smaller kernel bandwidths seem to produce more highly resolved patterns, the extreme variation in the coefficient estimates across bandwidths (Table 1) indicates a very high level of uncertainty. Since there is currently no scientific consensus on estimation of confidence intervals for GWR, we investigated the reliability of the GWR coefficient estimates for each bandwidth by computing estimates of standard error, using the bootstrapping technique [40], which were then expressed as a percentage of the coefficient estimates (Fig. 4, top: A, B). This showed that reducing the kernel size increased error in the coefficient estimates to an unacceptable degree (Fig. 4, top: A, B). For the 10000 m bandwidth models, estimated standard error is around 20% of the coefficient estimate values for three quarters of the data (18.82 for NO_2_ and 22.37 for PM_2.5_ at the 3rd quartile). Though already quite large, the boxplot (Fig. 4, top: A, B) shows how this error seems to increase sharply as the kernel bandwidth is reduced. Clearly, only the largest bandwidth (10000 m) is likely to provide anything approaching reliable estimates (75% of the data show <20% error). Unfortunately, the coefficient estimates obtained by the GWR model for this bandwidth (Fig. 4, bottom: C, D: left-hand maps for each contaminant), show only the broadest general pattern (stronger negative correlation in the north of the study area, where contamination levels are lowest). No useful conclusions can be drawn about the relationship between GHI and contamination levels on this basis.

### 3.3 Strategy 2: OLS results

Unlike Strategy 1, OLS carried out for data assembled under Strategy 2 shows no evidence of correlation between any of the NO_2_ aggregate variables or GHI. All models show very poor fit (NO_2_min ∼ GHI [R^2^ = 0.00, RSE = 8.60], NO_2_max ∼ GHI [R^2^ = 0.02, RSE = 8.36], NO_2_mean ∼ GHI [R^2^ = 0.00, RSE = 8.24], NO_2_sum ∼ GHI [R^2^ = 0.02, RSE = 91.1714]).

### 3.4 Strategy 2: SAR results

The spatial autoregressive model (SAR) highlights the relative unimportance of the GHI variable under strategy 2 compared to the presence of the contaminant in the neighborhood represented by the spatially lagged NO_2_ variable (Table 2). This holds true in all SAR models, including the poorly performing *NO*_*2*_*sum ∼ lagged NO*_*2*_*sum, GHI* model. Given the clear negative results and the similarity between NO_2_ and PM_2.5_ results under other data aggregation strategies, the Strategy 2 analysis was not carried out for PM_2.5_.

**Table 2:** Results of the spatial autoregressive (SAR) model for Nitrogen Dioxide (NO_2_) minimum, maximum, mean and sum against spatially lagged NO_2_ (using Queen's contiguity and immediate neighbours) and gross household income (GHI) per census tract.

Model	R^2^	RSE	DF	variable	coefficient	P value
NO_2_min ∼ lagged NO_2_min, GHI	0.96	1.73	3836	_	_	_
				*Lagged NO*_*2*_*min*	*9.66E-01*	*0*
				*GHI*	*5.31E-07*	*0.67*
NO_2_max ∼ lagged NO_2_max, GHI	0.86	3.14	3836	_	_	_
				*Lagged NO*_*2*_*max*	*0.93*	*0*
				*GHI*	*4.21E-06*	*0.07*
NO_2_mean ∼ lagged NO_2_mean, GHI	0.97	1.32	3836	_	_	_
				*Lagged NO*_*2*_*mean*	*0.99*	*0*
				*GHI*	*2.09E-06*	*0.03*
NO_2_sum ∼ lagged NO_2_sum, GHI	0.11	86.87	3836	_	_	_
				*Lagged NO*_*2*_*sum*	*0.28*	*0*
				*GHI*	*0.00039*	*0*

**Table 3:** Results of GWR analysis under Strategy 3, in comparison with results of OLS regression.

Analysis	model	Coef.	Coef. Min	Coef. Median	Coef. Max
Global OLS PM2.5	pm25min ∼ GHI	−0.00003			
	pm25max ∼ GHI	−0.00003			
	pm25mean ∼ GHI	−0.00003			
	pm25sum ∼ GHI	−0.00002			
GWR PM25 adaptive BW	_	_	−0.00059	0.00000	0.00085
Global OLS NO2	NO2min ∼ GHI	−0.00013			
	NO2max ∼ GHI	−0.00012			
	NO2mean ∼ GHI	−0.00012			
	NO2sum ∼ GHI	−0.00009			
GWR NO2 min BW 10000	_	_	−0.00019	−0.00007	0.00010
GWR NO2 min BW 5000	_	_	−0.00028	−0.00003	0.00020
GWR NO2 min BW 2500	_	_	−0.00062	−0.00001	0.00060
GWR NO2 min BW 1500	_	_	−0.00192	−0.00001	0.00130
GWR NO2 adaptive BW	_	_	−0.00125	0.00000	0.00471

As with the GWR analysis developed under Strategy 1, GWR results for the Strategy 3 dataset showed a greater range of variation in the regression coefficients than could be seen from the global OLS results (Table 3).

### 3.5 Strategy 3: OLS results

As with Strategy 1, but in contrast to Strategy 2, global level OLS regression indicated a negative correlation between level of household income and exposure to both NO_2,_ and PM_2.5_. The global regression models explained 15% and 19% of the variance for PM_2.5_ and GHI and between 11% and 13% of the variance for NO_2_ and GHI, depending on the summary statistic used (minimum, maximum, or mean). Results for sum had much lower explanatory power, but these models were found to violate the normality assumption for model residuals and can be safely discounted. The strongest association (highest coefficient of determination R^2^ and lowest residual standard error RSE) between income and air pollution was found for minimum NO_2_ and GHI and minimum PM_2.5_ and GHI, but all summary statistics except sum gave convincing, and highly similar, correlations. The best performing models in the OLS analysis, minimum NO_2_ ∼ GHI and minimum PM_2.5_ ∼ GHI, were retained for use in the subsequent GWR analysis.

### 3.6 Strategy 3: BiLISA results

All three variables (GHI, PM_2.5_ and NO_2_) were found to be highly spatially autocorrelated (p < 0.01) (Moran's I > 0.8; p < 0.01). The low precision of the OLS models described in the preceding section is likely to be at least partly due to the spatial autocorrelation phenomenon.

**Fig. 5:**
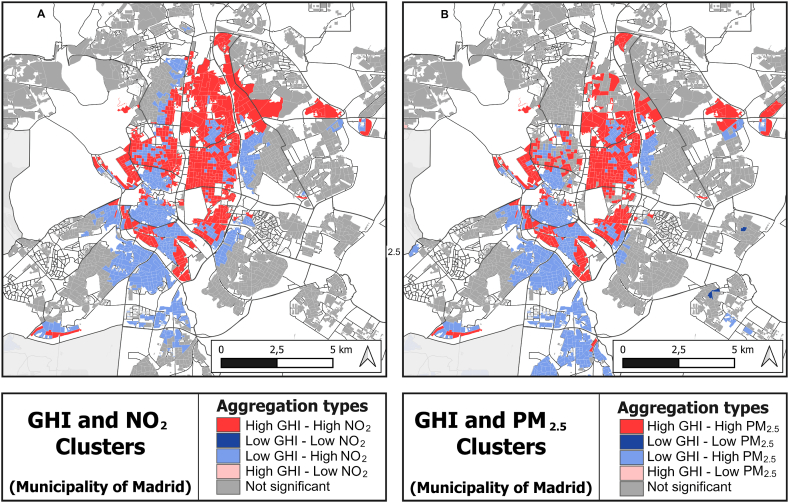
BiLISA results for NO_2_ (5A, left) and PM_2.5_ (5B, right) (minimum mean annual concentrations per spatial unit). (detailed view of Madrid centre).

Fig. 5 shows that the behaviour of both simulated pollutant concentrations versus GHI is very similar in both cases, creating clusters between high GHI and high pollutant concentration in the metropolitan area, clusters of high GHI and low pollutant concentration in the east and north of the study area and some in the east, clusters of low GHI and high pollutant concentrations in the south of the metropolitan centre and clusters of low GHI and low pollutant concentration in the urban areas in the periphery of the study area.

These clusters give interesting information about the behaviour of the two variables compared with each other. To the east there are urban areas of high GHI surrounded by other areas with low concentrations of pollutants. In the metropolitan area of Madrid there are areas with high GHI and high concentrations in the centre and towards the north, low GHI and high pollutant concentrations in the centre and expanding towards the south.

### 3.7 Strategy 3: geographically weighted regression (GWR) results

**Fig. 6:**
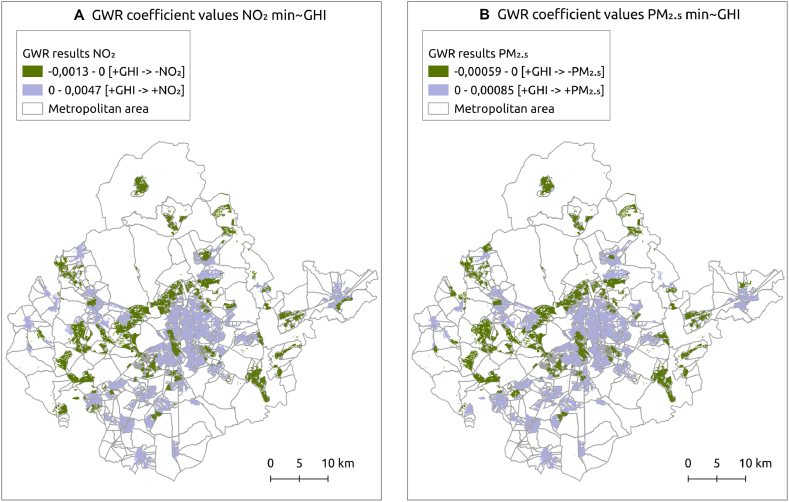
GWR results for NO_2_ (A: left) and PM_2.5_ (B: right). Negative coefficients (green) and Positive coefficients (mauve). Negative coefficients suggest that contamination may increase as income declines, while positive coefficients suggest that contamination may increase as income increases. Given the very small size of the coefficient estimates, these results should be treated with skepticism. (For interpretation of the references to colour in this figure legend, the reader is referred to the Web version of this article.)

Most importantly, the coefficients change sign, indicating that correlations between GHI and contaminants are negative at some locations (as income increases, exposure to air pollution decreases), and positive in others (as income increases, exposure to air pollution also increases) (Fig. 6). This supports the findings of the BiLISA analysis. However, the very small coefficient values indicate that the models are extremely sensitive to very small variations. SE could not even be estimated for the GWR models using the bootstrapping approach described earlier under Strategy 1, because coefficient values rounded to zero. This indicates that the results are highly unreliable despite the apparently acceptable model fit.

## 4 Discussion

The data analysed in this paper represent the best available information on household income not subject to statistical secrecy, and the largest-scale information available on air pollution for the two contaminants analysed, though this is derived from model simulations, and should not be confused with measurement data. Though both the city and autonomous region of Madrid maintain air pollution datasets at a very high temporal resolution derived from street level air quality monitoring stations, these data are not suitable for the detection of spatial patterns at the level of the district or street because the monitoring stations are too far apart. This analysis is a first attempt to address the important question of whether air pollution concentrations are correlated with household income based on available data. However, the task is a difficult one, due to the mismatch in scale and spatial unit size and shape between the km^2^ grid over which simulated contaminant concentrations are resolved, and the widely varying census tracts (*secciones censales*). Since income data are not available at a higher spatial resolution, larger census tracts inevitably include many km squares while the smallest may be entirely contained with a single square. In spatial terms, there is effectively no good solution, since information on contamination is lost within small census districts, while information on household income is lost in larger ones. We attempted to manage this using three different strategies, 1) one based on rasterizing the income data and aggregating the pixels into km^2^ units; 2) a second, which summarized the contamination data by census tract; and 3) a third approach, which we believe to be the most rigorous, in which the income data were assigned to residential areas before comparison with contamination data, thus eliminating the effect of large areas with no population from the spatial distribution of income. Nonetheless, while the study serves as a useful test case, none of these approaches were able to support the hypothesis that contamination is disproportionately a burden on those on lower incomes. Though the use of linear regression models might be criticized as simplistic, the very widely varying distribution of the data points under all three strategies did not suggest that different regression models, such as generalized additive models (see e.g. Ref. [21]) would produce better results; the coplots (Fig. 3) show this quite clearly. Given the uncertainty arising from the scale differences between the datasets, use of more sophisticated curve fitting approaches to find better regression fits would risk overfitting and the generation of quite spurious relationships. A further question is the effect of sample size, which is known to be important in regression modelling. The strongest evidence for a relationship between GHI and contamination was found in the smallest datasets (Strategy 1 datasets aggregated to the 4 km^2^ grid, n = 504). However, since statistical power is known to *increase* with sample size, we conclude that the only result which appeared to support the hypothesis is also statistically the weakest.

In addition to the difficulties related to mismatching size and shape of the spatial units of the datasets analysed, the relationship between incomes and air pollution in Madrid is anyway quite complicated. If we inspect the contamination and income maps side by side (Figs. 1 and 2), we can clearly see that the central northern area of the city of Madrid, coloured pale for the highest decile incomes, is also likely to be seriously affected by air pollution, above all, by NO_2_. By contrast, the districts of Usera and Villaverde, and parts of the municipalities of Getafe, Pinto and Parla would seem to be lower income areas with high contamination exposure (Fig. 6), The BiLISA analysis (Fig. 5) allows us to entertain this hypothesis, which is rendered unfortunately very uncertain by GWR analysis, which detects the same patterns but does not inspire confidence in them due to the extreme sensitivity of the model to tiny variations in coefficient values.

Thus, while the present study has not been able to provide statistical evidence to support the hypothesis that air pollution disproportionately affects those on lower incomes, we do not feel that this hypothesis should be abandoned just yet. Madrid is a highly unequal city. A diagonal line drawn from the A5 north of Mostoles in the southwest to the historic town of Alcalá de Henares in the east leaves most of Madrid's low-income neighbourhoods on one side (south) and most of Madrid's high-income neighbourhoods on the other (north). This division can be easily appreciated in Fig. 2. Lower socioeconomic status (on several measures including income) in Madrid has been found to be correlated with increased prevalence of particular diseases, for example diabetes [41] The city's high air pollution burden is also known to affect some demographic groups disproportionately – [17] found that older people (>65 years) were over-exposed to NO_2_ pollution compared to the population average, since they are over-represented in inner city neighbourhoods. Elsewhere, several studies have noted a higher exposure to pollutants in lower income districts or counties (e.g. [42-3]), the phenomenon is particularly well-demonstrated in the US [44]. These studies indicate a number of future promising lines of enquiry. It might be helpful to extend the rather simplistic income indicator to a generalized socioeconomic deprivation index, e.g. including education level, and employment status, or substitute the GHI statistic for different measure of wealth, such as average house prices per sq. m. (see e.g. Ref. [41]). Some studies have shown ethnicity to be more strongly associated with increased air pollution exposure than poverty (e.g. Ref. [43]), something that could be tested for the case of Madrid.

Given the high level of uncertainty arising from the various data aggregation strategies, as well as the use of simulated concentrations rather than measurement data, more attention should be also paid to data quality and resolution. The problems caused by the rather sparse network of air quality monitoring stations could be resolved by systematic collection of high spatial resolution air pollution data in areas of interest, for example across a grid transect including the northern part of the city of Madrid (Calle Serrano and the Castellana) and zones immediately west and east (an east-west long axis), and a second grid transect with a north-south long-axis crossing the north-south boundaries of the districts of Usera and Villaverde out to Getafe and Pinto to the south. Under such an approach, mobile air pollution sensors could be deployed in these two target areas to collect data on a grid of 100 m, for example, enabling the hypothesis to the explored without the high cost of mapping the whole of the city. Household level socioeconomic statistics could be acquired, if the necessary permissions can be obtained, or collected through telephone or internet surveys, allowing for a more complete picture to be developed of these two contrasting areas. Of particular interest is the study by Ref. [45], which used mobility information based on mobile phone data to assess air pollution exposure at different times of day. Mobile phone information can be used to estimate income statistics (se e.g. Ref. [46]) which, combined with the higher resolution information on individual mobility that these data already provide, would potentially allow for a mobility-based study of income and air pollution exposure. Not only would such a study help provide an answer to the question of differential exposure by income based on residential location, which is widely documented elsewhere, but it would also help to understand how air pollution exposure changes depending on individual mobility. Since the ability to work remotely is unequally distributed, disproportionately favouring higher-skilled white-collar workers [47], lower-skilled workers on lower incomes in the service economy may commute greater distances, thus accumulating more exposure to air pollution.

## 5 Conclusions

Our study explored the hypothesis that air pollution disproportionately affects lower income households using the case study of Madrid, Spain. Three different strategies were adopted to harmonize data and to overcome problems of mismatch in spatial scales and size of spatial units. Strategies 1 and 3 suggested a correlation between level of household income and exposure to both pollutants, though with very high variance and weak explanatory power (10–18% of the variance explained). GWR results for Strategy 1 suggested a stronger relationship between contaminants and income in the north of the study area, but bootstrap estimates of standard error indicated low confidence in the results, with error increasing to >100% with smaller bandwidths. For Strategy 2, linear models had virtually no explanatory power, though this was probably due to the mismatch between the census tracts and km^2^ grids. Not surprisingly the spatial regressions carried out under Strategy 2 found that simulated concentrations of NO_2_ in census tracts were mostly explained by simulated concentrations of NO_2_ in neighboring census tracts. For Strategy 3, which we considered the most robust, linear models showed some explanatory power, with considerably improved model fit under GWR, with some locations showing negative correlations between GHI and contaminants (as income increases, exposure to air pollution decreases), and others showing positive correlations (as income increases, exposure to air pollution also increases). Unfortunately, the effect size was extremely small, meaning that reliable conclusions cannot be drawn.

Our study highlights the potential usefulness of electoral district level income data for evaluating environmental inequality, but also illustrates the major disadvantages that such data bring when trying to compare against data obtained on a regular grid. We find that statistical model fit is extremely sensitive to the data processing strategy adopted, which should offer a cautionary tale for similar studies. Though literature suggests that air pollution is likely to disproportionately affect lower-income populations, with these data, in this study area, we were not able to confirm this hypothesis. Our study and the recommendations arising from it have important implications for the monitoring, data collection, modeling and statistical analysis of air pollution and its socioeconomic impacts worldwide.

## Data and codes availability statement

The data and codes that support the findings of this study and allow the results to be reproduced are available at: Hewitt et al. Is air pollution exposure linked to household income? Spatial analysis of Community Multiscale Air Quality Model results for Madrid (Data and scripts) (figshare.com).

## Ethics declarations

Review and/or approval by an ethics committee was not needed for this study because no work was conducted on human or animal subjects and no personal information was obtained from the population data used.

## CRediT authorship contribution statement

**Richard J. Hewitt:** Writing – review & editing, Writing – original draft, Project administration, Methodology, Investigation, Funding acquisition, Formal analysis. **Eduardo Caramés:** Writing – original draft, Visualization, Investigation, Formal analysis. **Rafael Borge:** Writing – review & editing, Supervision, Data curation, Conceptualization.

## Declaration of competing interest

The authors declare that they have no known competing financial interests or personal relationships that could have appeared to influence the work reported in this paper.

## Footnotes

1 Piwowar suggests that “the grid cell size should be one-fourth of the area of the MMU in order to maintain the integrity of [the] data” [31]. Excepting outliers, the smallest census tracts in the study area were c. 9000 m^2^. This gives us a grid cell size of 9000/4 = 2250 m^2^, or, approximating to the nearest even number, a regular square cell of 48x48 m.
