# Supplementary material for: Is air pollution exposure linked to household income? Spatial analysis of Community Multiscale Air Quality Model results for Madrid
Source: Heliyon. 2024 Feb 24;10(5):e27117. doi: 10.1016/j.heliyon.2024.e27117 (PMC10909769; doi:10.1016/j.heliyon.2024.e27117)

**Is air pollution exposure linked to household income? Spatial analysis of Community Multiscale Air Quality Model results for Madrid.**

**Supplementary material**

**1. CMAQ model operational evaluation procedures and model performance**

The Community Multiscale Air Quality (CMAQ) model (Byun and Ching, 1999, Byun and Schere, 2006) is a state-of-the-science chemical-transport model widely used by the air quality community. It has been applied all over the world, including applications to study urban air pollution at high resolution in many cities, Madrid among them. Most of those studies include a comparison of model predictions with observations from the local air quality monitoring network to assess model performance that demonstrate a satisfactory performance for this kind of tools (Borge et al., 2018, 2014; 2012; de la Paz et al., 2016; Mircea et al., 2023; Saiz-Lopez et al., 2017; Vedrenne et al., 2016).

A comprehensive overview of the diagnostic value of the simulation that supports this specific simulation can be found in **Borge et al. (2022)**. The model performance analysis is performed in the framework of typical operational evaluation techniques (Dennis et al., 2010) for mesoscale air quality models using all available information (hourly concentration records of the main pollutants, including NO_2_ and PM_2.5_ for the entire year 2015) from all the monitoring stations in the region (47 and 14, respectively). A detailed discussion of the overall model performance, including a stratification by monitoring site type is available in the supplementary material of Borge et al. (2022). From that statistical analysis, the authors conclude that the model performance is in the usual range of mesoscale modelling systems with a global normalized bias of -24% and -3% and Pearson correlation coefficient of 0,71 and 0,35 for NO_2_ and PM_2.5_, respectively (Table SM1). More importantly, a consistent performance throughout the territory was found, suggesting that the model is able to accurately represent pollution spatial gradients that are essential for the validity of our research.


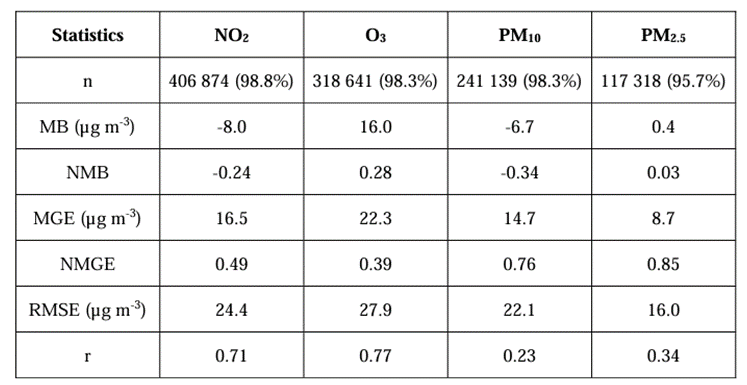


Table SM1. Model performance summary (from Borge et al., 2022)

**References**

Borge et al., 2012. Atmospheric Environment 62, 461–471. <https://doi.org/10.1016/J.ATMOSENV.2012.08.073>

Borge et al., 2014. Science of The Total Environment 466–467, 809–819. <https://doi.org/10.1016/j.scitotenv.2013.07.093>

Borge et al., 2018. Science of The Total Environment 635, 1574–1584. <https://doi.org/10.1016/J.SCITOTENV.2018.04.323>

Borge et al., 2022. Atmospheric Environment 287, 119258. <https://doi.org/10.1016/j.atmosenv.2022.119258>

Borge et al., 2023. Forests 14(6), 1255. <https://doi.org/10.1016/j.atmosenv.2022.119258>

Byun, D., Schere, K.L., 2006. Applied Mechanics Reviews 59(2): 51-77. <https://doi.org/10.1115/1.2128636>

Byun, D.W., Ching, J.K.S., 1999. <https://www.cmascenter.org/cmaq/science_documentation/pdf/ch01.pdf>

de la Paz, 2016. Atmospheric Environment 144, 282–296. <https://doi.org/10.1016/J.ATMOSENV.2016.08.082>

Dennis et al., 2010. Environmental Fluid Mechanics 10, 471–489. <https://doi.org/10.1007/s10652-009-9163-2>

Mircea et al., 2023. Forests 14(6), 1255. <https://doi.org/10.3390/f14061255>

Saiz-Lopez et al., 2017. Scientific Reports 7, 1–11. <https://doi.org/10.1038/srep45956>

Vedrenne et al., 2016. Atmospheric Environment 145, 29–44. <https://doi.org/10.1016/J.ATMOSENV.2016.09.020>

**2. Table of Results for Strategy 1 Ordinary Least Squares regression.**

| **No.** | **Analysis** | **Model** | **R2** | **RSE** | **dfree** | **F.statistic** | **P.value** | **sample_size** | **Figure (below)** |
| --- | --- | --- | --- | --- | --- | --- | --- | --- | --- |
| 1 | NO2renta1_1_100clip | yvar ~ xmin | 0.137 | 0.569 | 1878 | 298 | 0 | 1880 | SM1 |
| 2 | NO2renta1_1_100clip | yvar ~ xmax | 0.011 | 0.609 | 1878 | 21.5 | 0 | 1880 | SM1 |
| 3 | NO2renta1_1_100clip | yvar ~ xmean | 0.053 | 0.596 | 1878 | 106 | 0 | 1880 | SM1 |
| 4 | NO2renta1_1_100clip | yvar ~ xsum | 0.052 | 0.596 | 1878 | 104 | 0 | 1880 | SM1 |
| 5 | NO2renta1_1_200clip | yvar ~ xmin | 0.127 | 0.572 | 1878 | 273 | 0 | 1880 | SM2 |
| 6 | NO2renta1_1_200clip | yvar ~ xmax | 0.013 | 0.608 | 1878 | 24 | 0 | 1880 | SM2 |
| 7 | NO2renta1_1_200clip | yvar ~ xmean | 0.053 | 0.596 | 1878 | 105 | 0 | 1880 | SM2 |
| 8 | NO2renta1_1_200clip | yvar ~ xsum | 0.049 | 0.597 | 1878 | 96.6 | 0 | 1880 | SM2 |
| 9 | NO2renta1_1_48clip | yvar ~ xmin | 0.14 | 0.568 | 1878 | 306 | 0 | 1880 | SM3 |
| 10 | NO2renta1_1_48clip | yvar ~ xmax | 0.012 | 0.609 | 1878 | 22.4 | 0 | 1880 | SM3 |
| 11 | NO2renta1_1_48clip | yvar ~ xmean | 0.054 | 0.596 | 1878 | 106 | 0 | 1880 | SM3 |
| 12 | NO2renta1_1_48clip | yvar ~ xsum | 0.053 | 0.596 | 1878 | 105 | 0 | 1880 | SM3 |
| 13 | NO2renta1_1_500clip | yvar ~ xmin | 0.103 | 0.58 | 1878 | 217 | 0 | 1880 | SM4 |
| 14 | NO2renta1_1_500clip | yvar ~ xmax | 0.021 | 0.606 | 1878 | 40.7 | 0 | 1880 | SM4 |
| 15 | NO2renta1_1_500clip | yvar ~ xmean | 0.053 | 0.596 | 1878 | 105 | 0 | 1880 | SM4 |
| 16 | NO2renta1_1_500clip | yvar ~ xsum | 0.05 | 0.597 | 1878 | 98 | 0 | 1880 | SM4 |
| 17 | NO2renta2_2_100clip | yvar ~ xmin | 0.196 | 0.548 | 502 | 122 | 0 | 504 | SM5 |
| 18 | NO2renta2_2_100clip | yvar ~ xmax | 0 | 0.611 | 502 | 0 | 0.98 | 504 | SM5 |
| 19 | NO2renta2_2_100clip | yvar ~ xmean | 0.044 | 0.597 | 502 | 23.3 | 0 | 504 | SM5 |
| 20 | NO2renta2_2_100clip | yvar ~ xsum | 0.042 | 0.598 | 502 | 22.3 | 0 | 504 | SM5 |
| 21 | NO2renta2_2_200clip | yvar ~ xmin | 0.18 | 0.553 | 502 | 110 | 0 | 504 | SM6 |
| 22 | NO2renta2_2_200clip | yvar ~ xmax | 0 | 0.611 | 502 | 0.08 | 0.78 | 504 | SM6 |
| 23 | NO2renta2_2_200clip | yvar ~ xmean | 0.043 | 0.598 | 502 | 22.5 | 0 | 504 | SM6 |
| 24 | NO2renta2_2_200clip | yvar ~ xsum | 0.042 | 0.598 | 502 | 22.1 | 0 | 504 | SM6 |
| 25 | NO2renta2_2_48clip | yvar ~ xmin | 0.188 | 0.551 | 502 | 116 | 0 | 504 | SM7 |
| 26 | NO2renta2_2_48clip | yvar ~ xmax | 0 | 0.611 | 502 | 0.05 | 0.83 | 504 | SM7 |
| 27 | NO2renta2_2_48clip | yvar ~ xmean | 0.044 | 0.598 | 502 | 22.9 | 0 | 504 | SM7 |
| 28 | NO2renta2_2_48clip | yvar ~ xsum | 0.043 | 0.598 | 502 | 22.5 | 0 | 504 | SM7 |
| 29 | NO2renta2_2_500clip | yvar ~ xmin | 0.17 | 0.557 | 502 | 103 | 0 | 504 | SM8 |
| 30 | NO2renta2_2_500clip | yvar ~ xmax | 0.004 | 0.61 | 502 | 2.17 | 0.14 | 504 | SM8 |
| 31 | NO2renta2_2_500clip | yvar ~ xmean | 0.046 | 0.597 | 502 | 24.4 | 0 | 504 | SM8 |
| 32 | NO2renta2_2_500clip | yvar ~ xsum | 0.049 | 0.596 | 502 | 25.6 | 0 | 504 | SM8 |
| 33 | PM25renta1_1_100clip | yvar ~ xmin | 0.139 | 0.294 | 1878 | 303 | 0 | 1880 | SM9 |
| 34 | PM25renta1_1_100clip | yvar ~ xmax | 0.059 | 0.307 | 1878 | 117 | 0 | 1880 | SM9 |
| 35 | PM25renta1_1_100clip | yvar ~ xmean | 0.096 | 0.301 | 1878 | 200 | 0 | 1880 | SM9 |
| 36 | PM25renta1_1_100clip | yvar ~ xsum | 0.094 | 0.302 | 1878 | 195 | 0 | 1880 | SM9 |
| 37 | PM25renta1_1_200clip | yvar ~ xmin | 0.135 | 0.295 | 1878 | 294 | 0 | 1880 | SM10 |
| 38 | PM25renta1_1_200clip | yvar ~ xmax | 0.06 | 0.307 | 1878 | 119 | 0 | 1880 | SM10 |
| 39 | PM25renta1_1_200clip | yvar ~ xmean | 0.097 | 0.301 | 1878 | 201 | 0 | 1880 | SM10 |
| 40 | PM25renta1_1_200clip | yvar ~ xsum | 0.09 | 0.302 | 1878 | 185 | 0 | 1880 | SM10 |
| 41 | PM25renta1_1_48clip | yvar ~ xmin | 0.141 | 0.294 | 1878 | 308 | 0 | 1880 | SM11 |
| 42 | PM25renta1_1_48clip | yvar ~ xmax | 0.06 | 0.307 | 1878 | 120 | 0 | 1880 | SM11 |
| 43 | PM25renta1_1_48clip | yvar ~ xmean | 0.097 | 0.301 | 1878 | 201 | 0 | 1880 | SM11 |
| 44 | PM25renta1_1_48clip | yvar ~ xsum | 0.096 | 0.301 | 1878 | 199 | 0 | 1880 | SM11 |
| 45 | PM25renta1_1_500clip | yvar ~ xmin | 0.121 | 0.297 | 1878 | 258 | 0 | 1880 | SM12 |
| 46 | PM25renta1_1_500clip | yvar ~ xmax | 0.066 | 0.306 | 1878 | 134 | 0 | 1880 | SM12 |
| 47 | PM25renta1_1_500clip | yvar ~ xmean | 0.095 | 0.301 | 1878 | 197 | 0 | 1880 | SM12 |
| 48 | PM25renta1_1_500clip | yvar ~ xsum | 0.084 | 0.303 | 1878 | 171 | 0 | 1880 | SM12 |
| 49 | PM25renta2_2_100clip | yvar ~ xmin | 0.185 | 0.286 | 502 | 114 | 0 | 504 | SM13 |
| 50 | PM25renta2_2_100clip | yvar ~ xmax | 0.027 | 0.313 | 502 | 13.7 | 0 | 504 | SM13 |
| 51 | PM25renta2_2_100clip | yvar ~ xmean | 0.09 | 0.302 | 502 | 49.4 | 0 | 504 | SM13 |
| 52 | PM25renta2_2_100clip | yvar ~ xsum | 0.087 | 0.303 | 502 | 47.9 | 0 | 504 | SM13 |
| 53 | PM25renta2_2_200clip | yvar ~ xmin | 0.176 | 0.288 | 502 | 107 | 0 | 504 | SM14 |
| 54 | PM25renta2_2_200clip | yvar ~ xmax | 0.03 | 0.312 | 502 | 15.4 | 0 | 504 | SM14 |
| 55 | PM25renta2_2_200clip | yvar ~ xmean | 0.087 | 0.303 | 502 | 48.1 | 0 | 504 | SM14 |
| 56 | PM25renta2_2_200clip | yvar ~ xsum | 0.086 | 0.303 | 502 | 47.3 | 0 | 504 | SM14 |
| 57 | PM25renta2_2_48clip | yvar ~ xmin | 0.18 | 0.287 | 502 | 110 | 0 | 504 | SM15 |
| 58 | PM25renta2_2_48clip | yvar ~ xmax | 0.029 | 0.312 | 502 | 15.1 | 0 | 504 | SM15 |
| 59 | PM25renta2_2_48clip | yvar ~ xmean | 0.089 | 0.303 | 502 | 48.8 | 0 | 504 | SM15 |
| 60 | PM25renta2_2_48clip | yvar ~ xsum | 0.088 | 0.303 | 502 | 48.2 | 0 | 504 | SM15 |
| 61 | PM25renta2_2_500clip | yvar ~ xmin | 0.161 | 0.29 | 502 | 96 | 0 | 504 | SM16 |
| 62 | PM25renta2_2_500clip | yvar ~ xmax | 0.046 | 0.31 | 502 | 24.3 | 0 | 504 | SM16 |
| 63 | PM25renta2_2_500clip | yvar ~ xmean | 0.092 | 0.302 | 502 | 50.8 | 0 | 504 | SM16 |
| 64 | PM25renta2_2_500clip | yvar ~ xsum | 0.09 | 0.302 | 502 | 49.8 | 0 | 504 | SM16 |

**3. OLS regression results (including confidence and prediction intervals)**

In the plots below (Figures SM1 to SM16). Confidence Intervals are shown as a grey band between higher and lower bounds, while prediction Intervals are shown as red dashed lines.

Figure SM1: NO2renta1_1_100clip


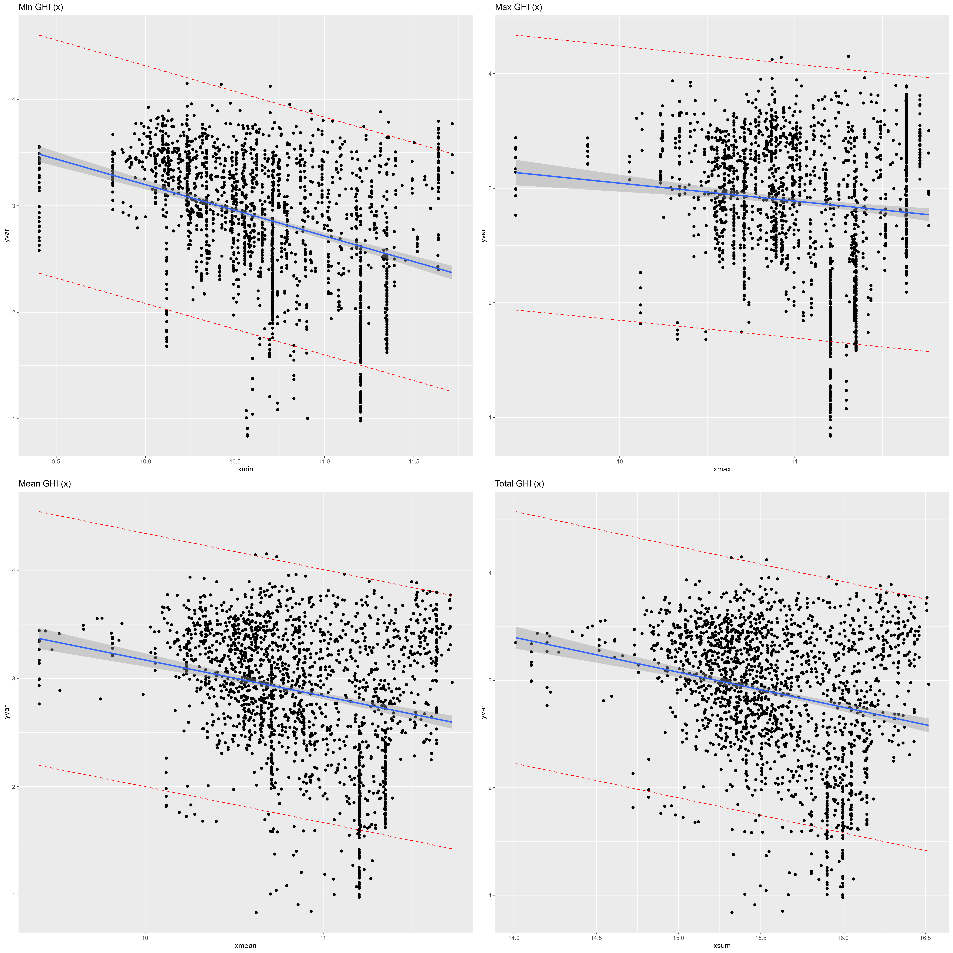


Figure SM2: NO2renta1_1_200clip


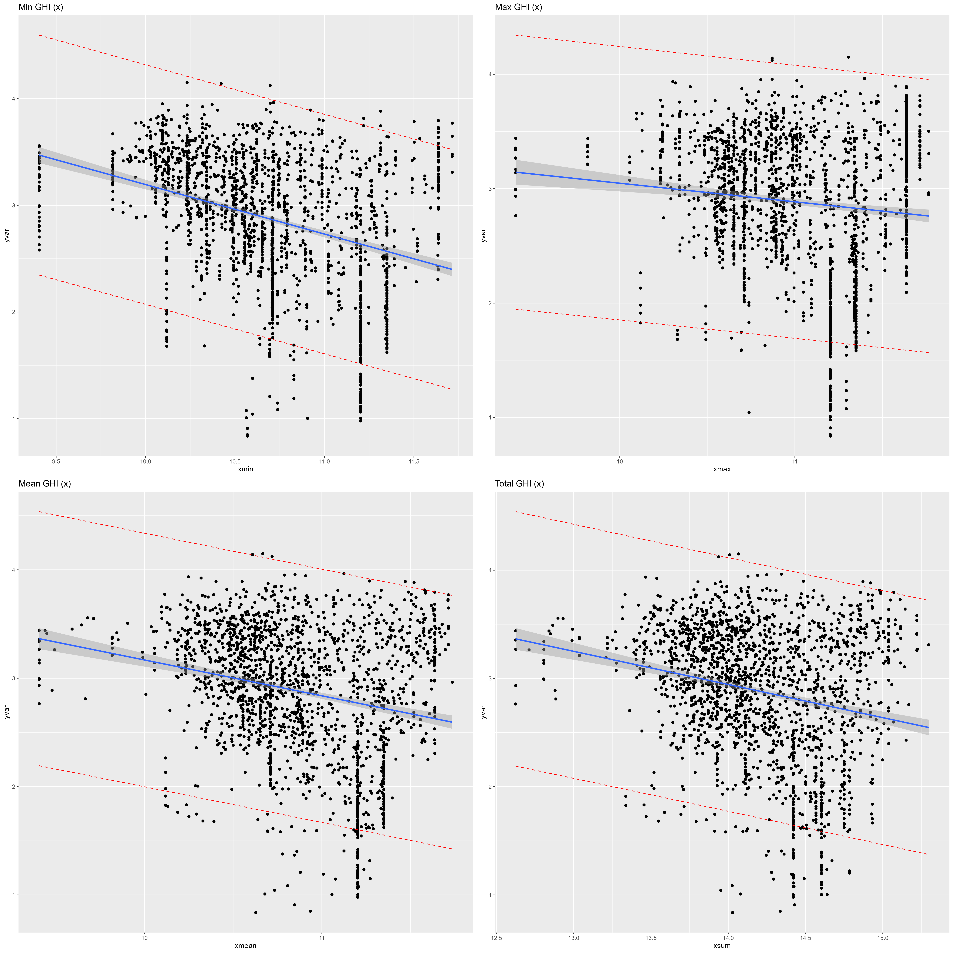


Figure SM3: NO2renta1_1_48clip


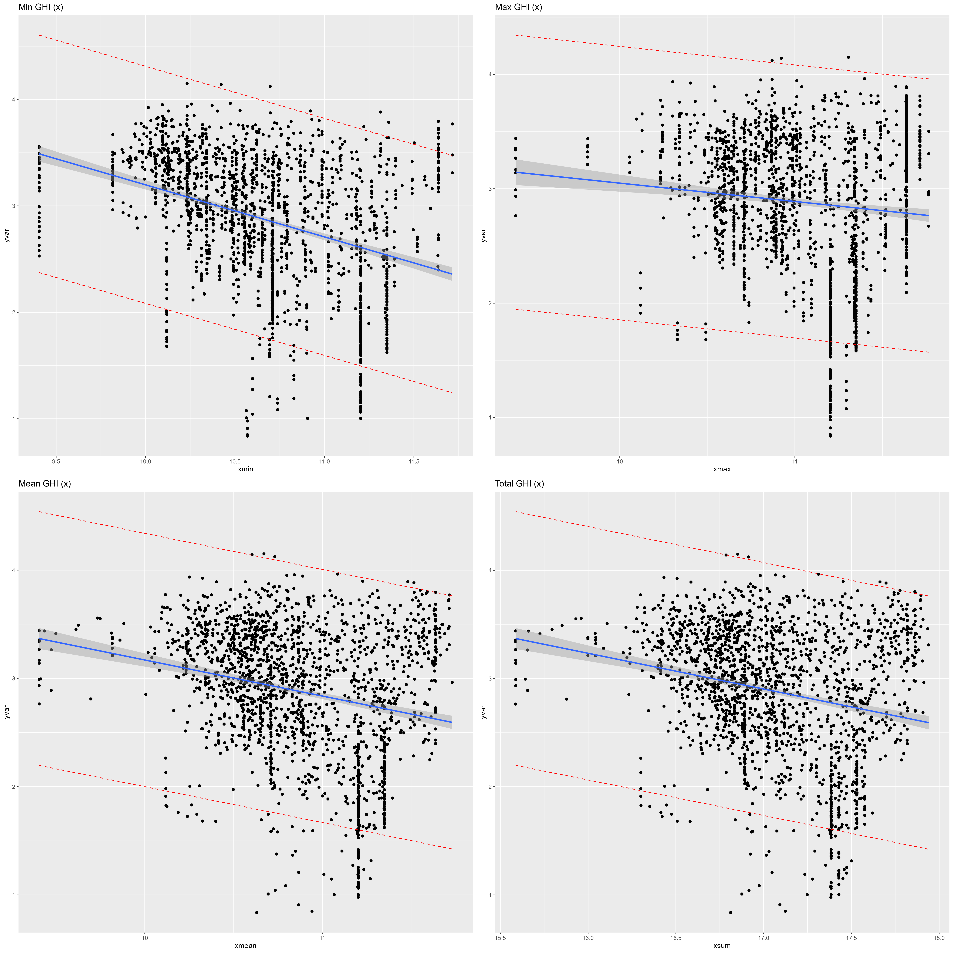


Figure SM4: NO2renta1_1_500clip


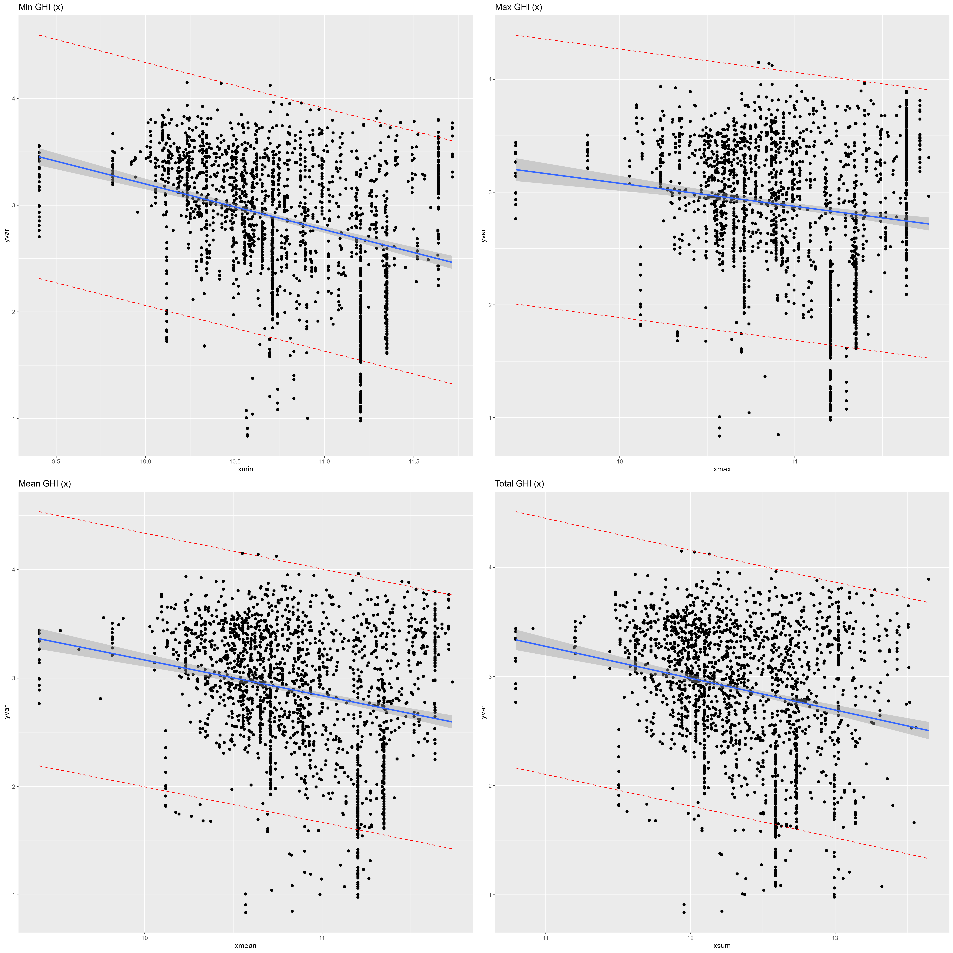


Figure SM5: NO2renta2_2_100clip


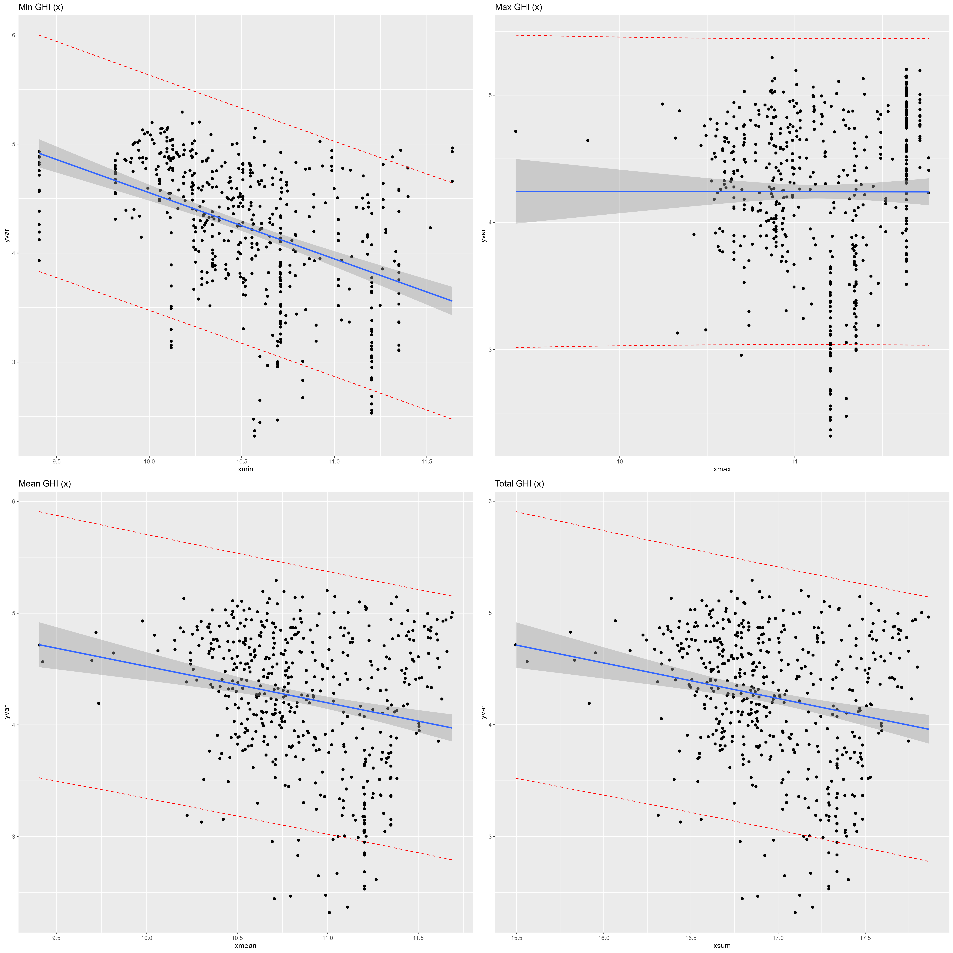


Figure SM6: NO2renta2_2_200clip


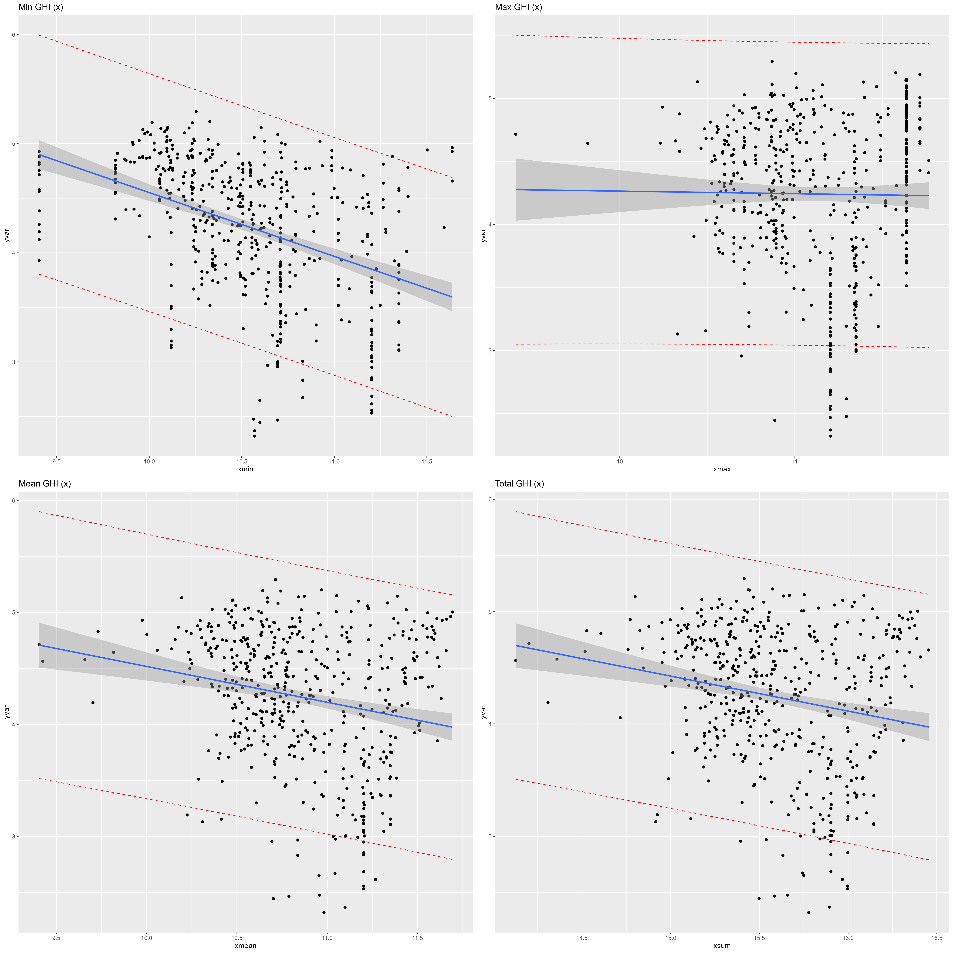


Figure SM7: NO2renta2_2_48clip


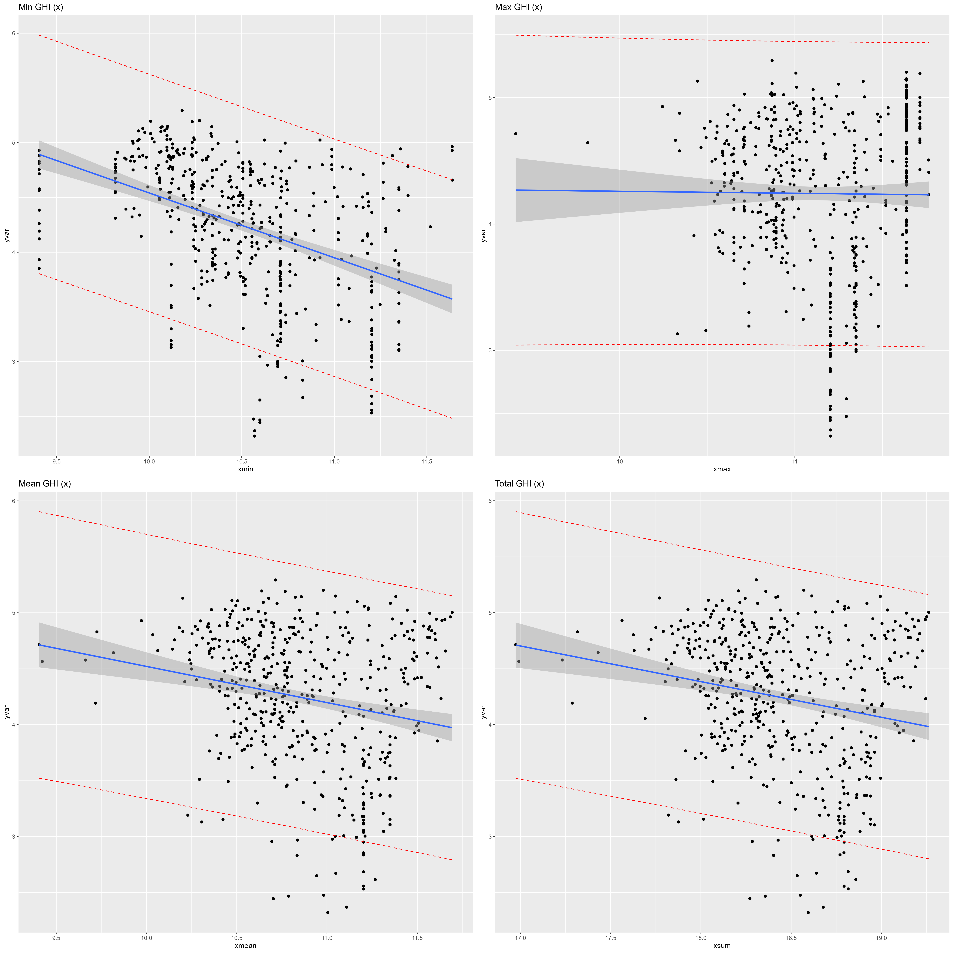


Figure SM8: NO2renta2_2_500clip


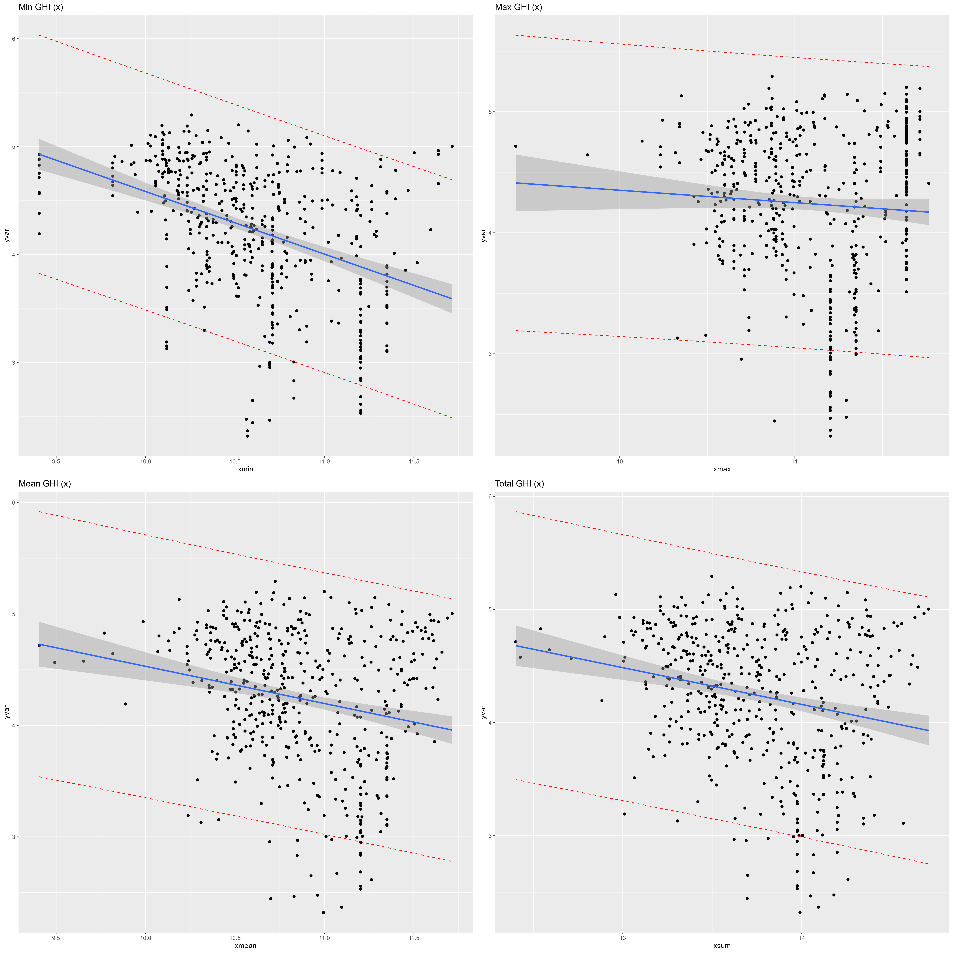


Figure SM9: PM25renta1_1_100clip


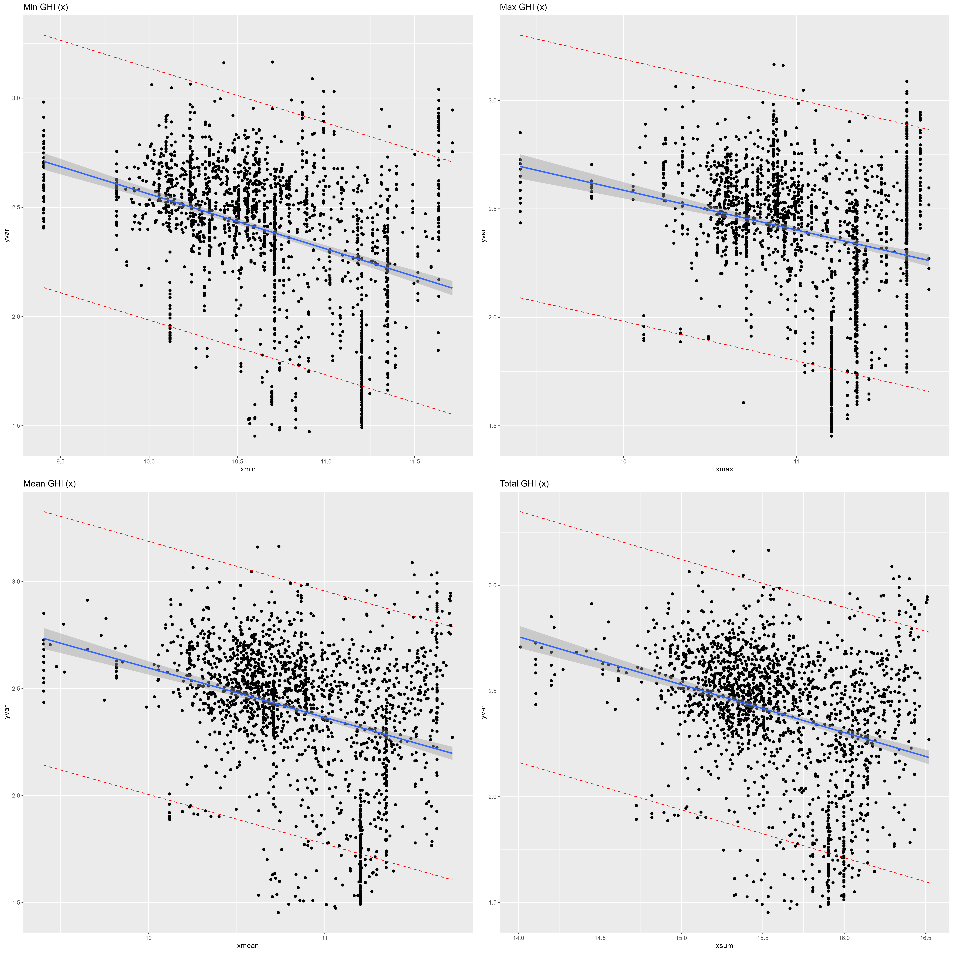


Figure SM10: PM25renta1_1_200clip


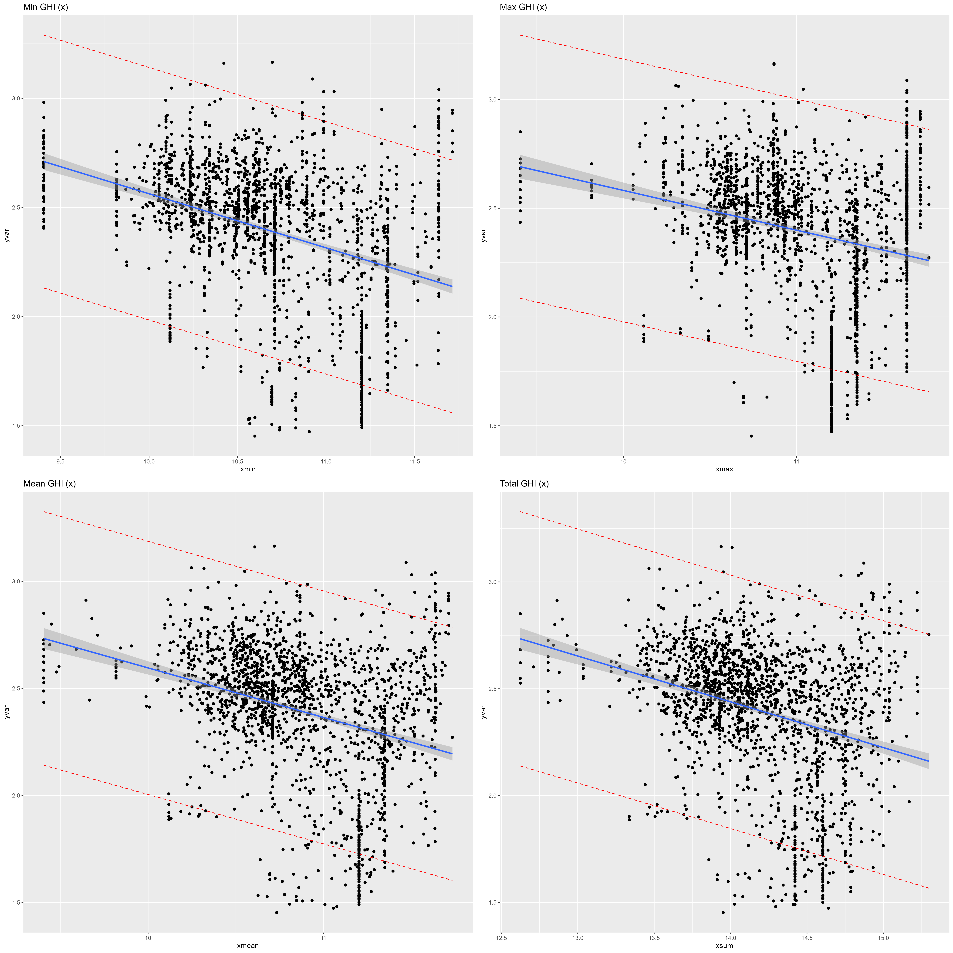


Figure SM11: PM25renta1_1_48clip


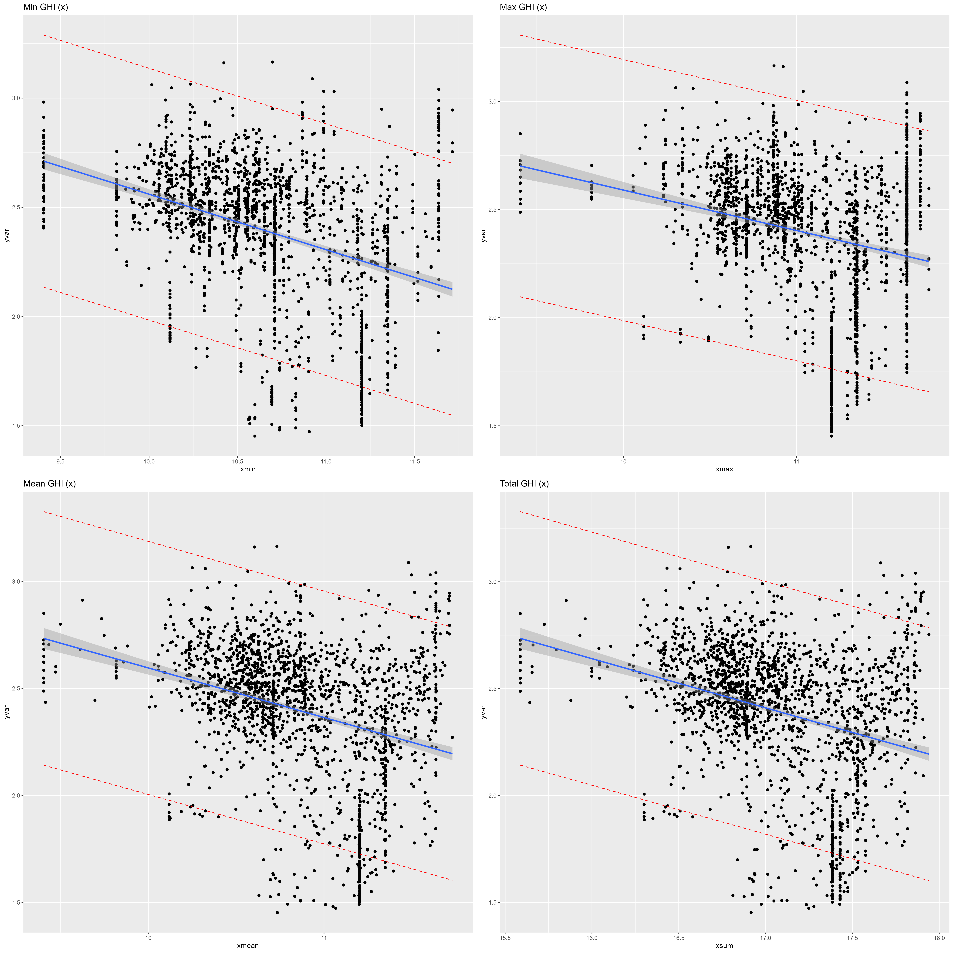


Figure SM12: PM25renta1_1_500clip


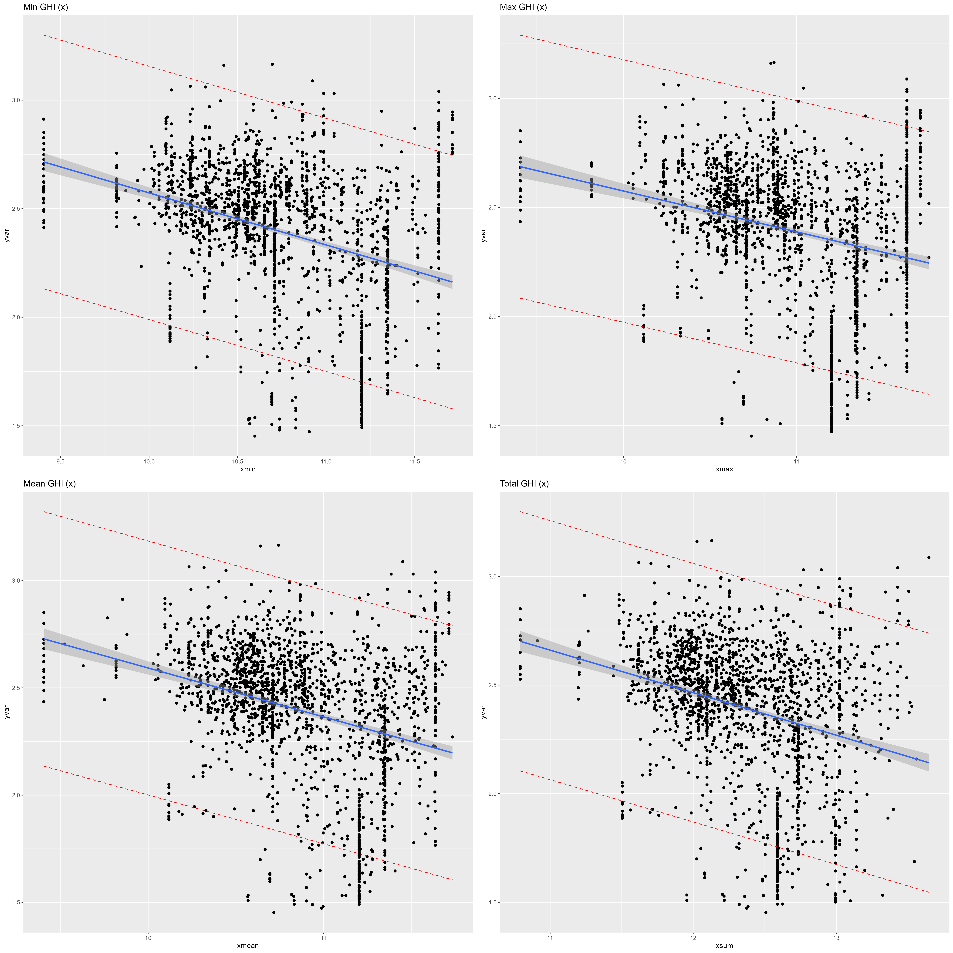


Figure SM13: PM25renta2_2_100clip


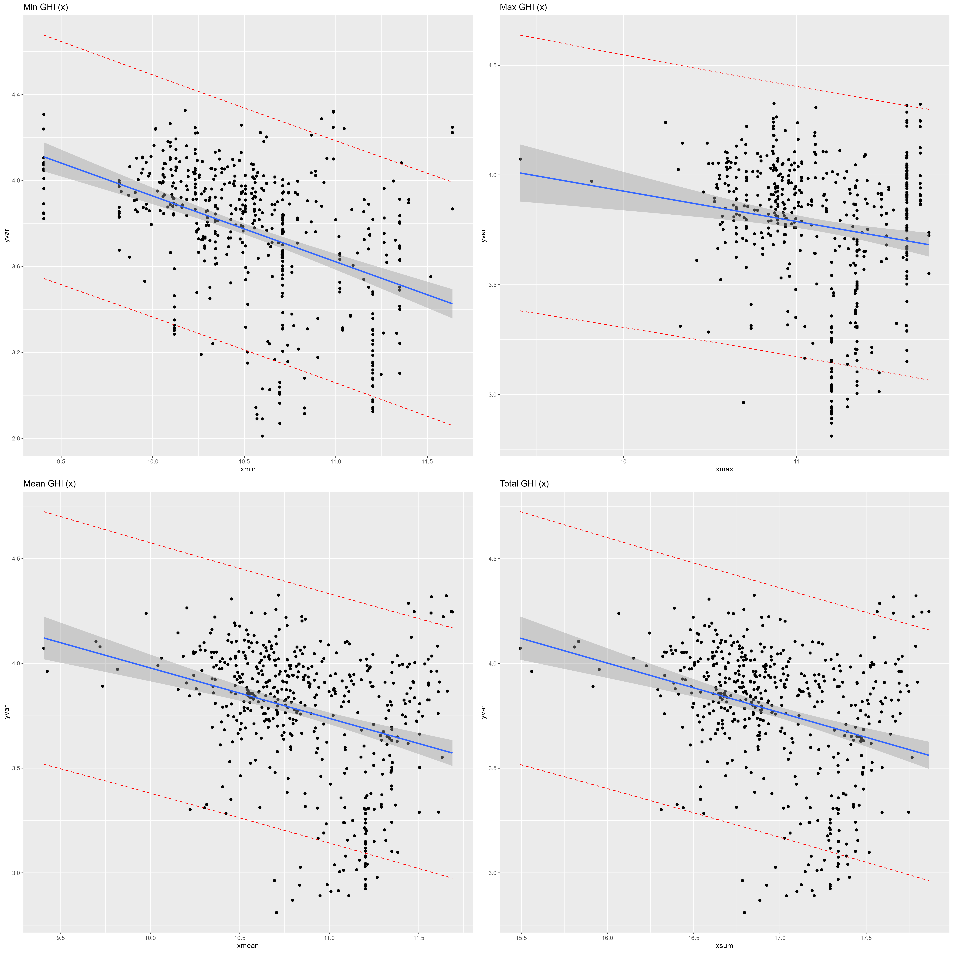


Figure SM14: PM25renta2_2_200clip


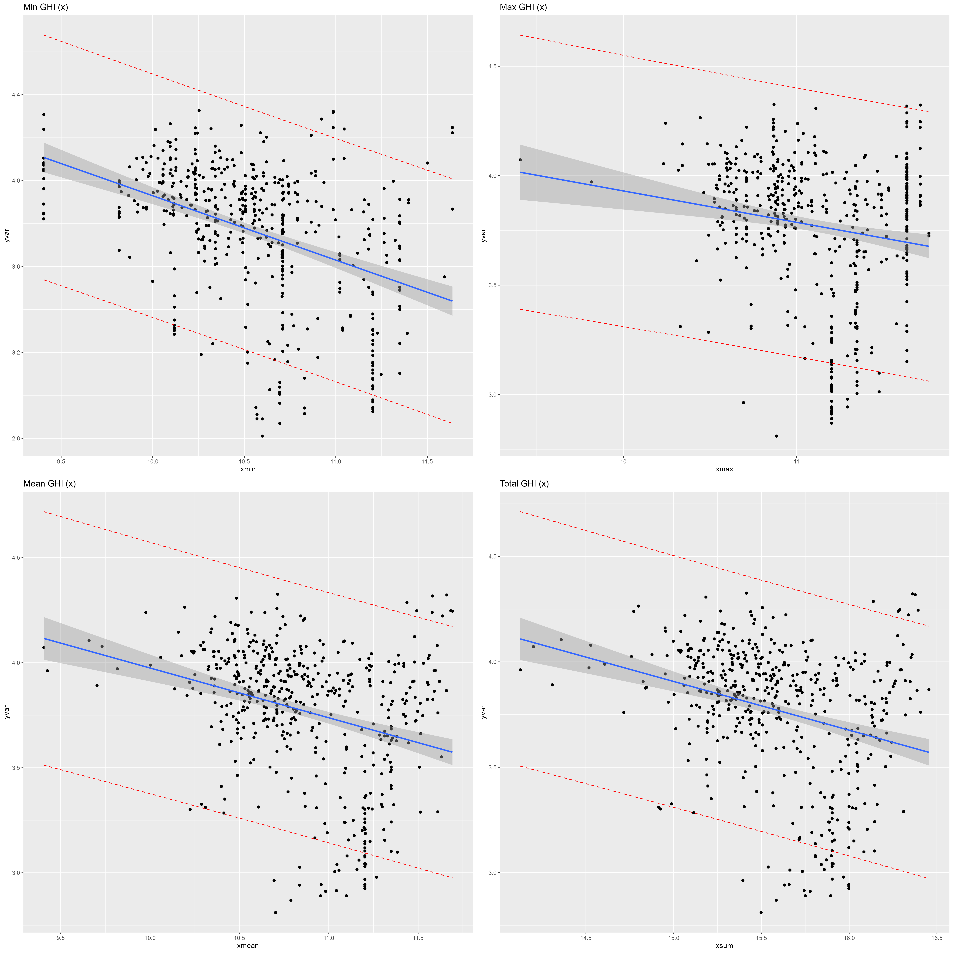


Figure SM15: PM25renta2_2_48clip


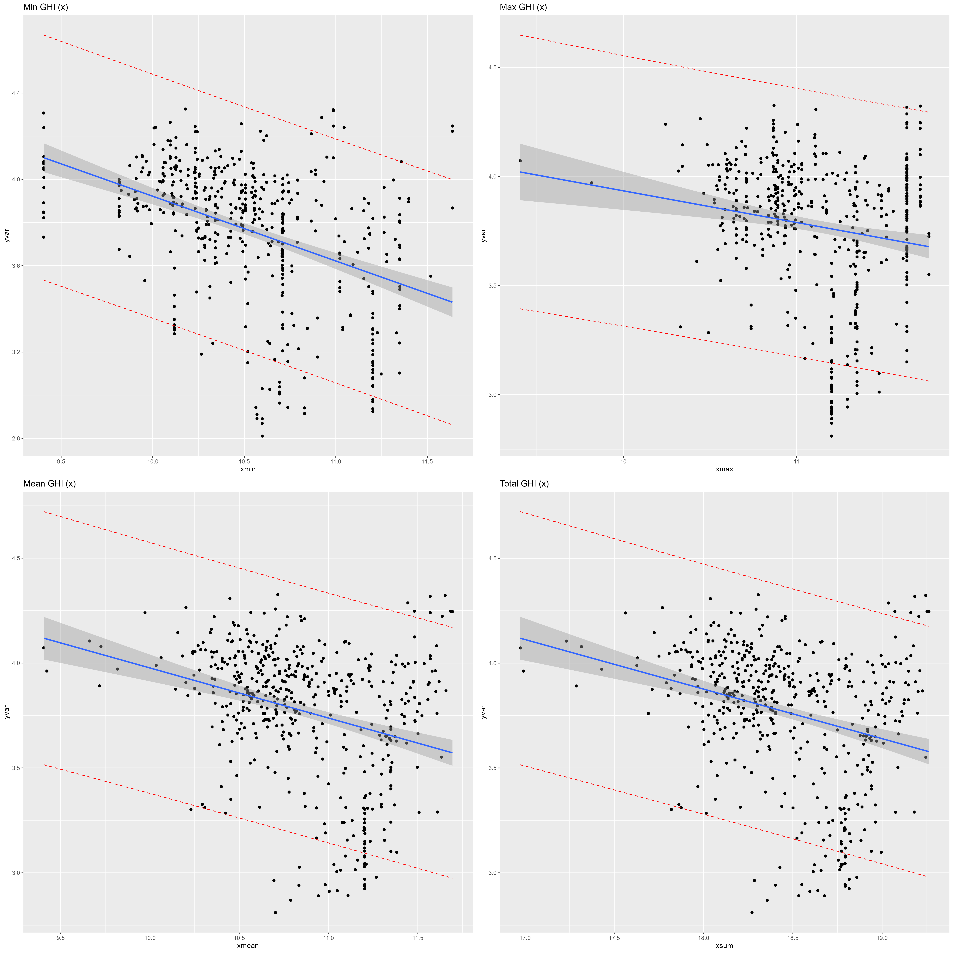


Figure SM16: PM25renta2_2_500clip


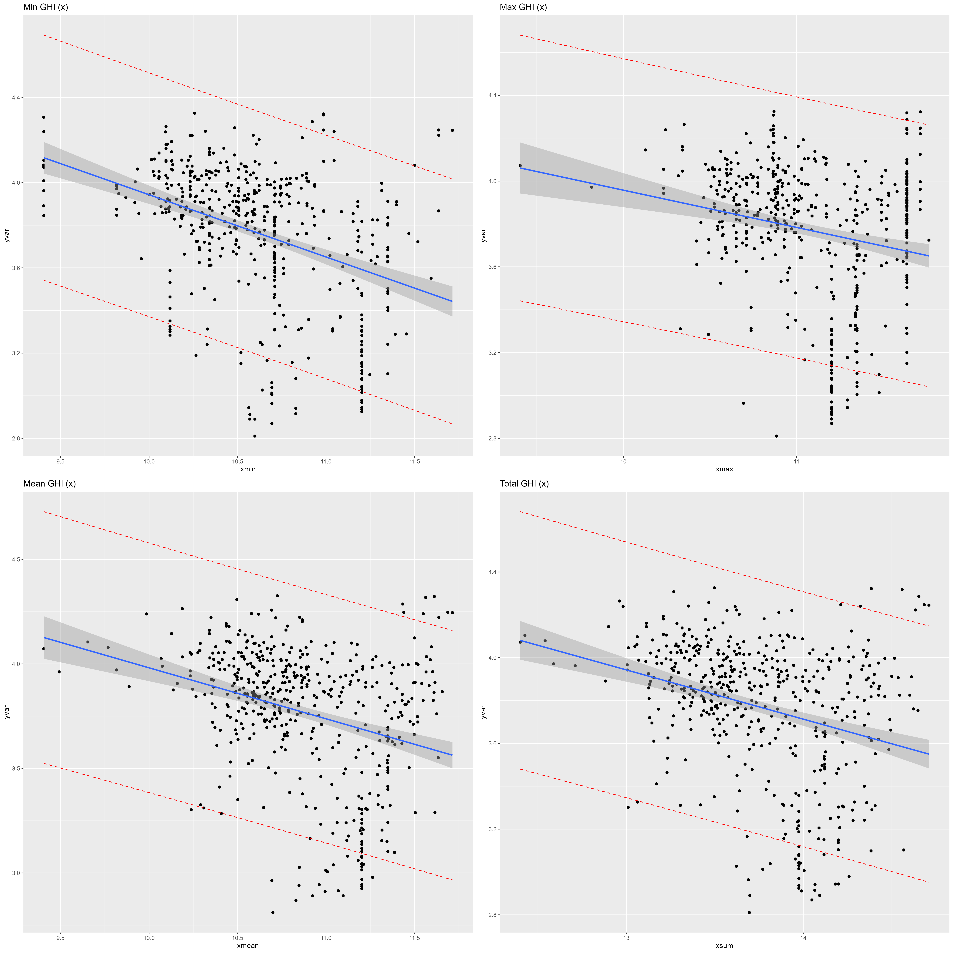

Supplement: Multimedia component 1 [file mmc1.docx]
